# Interventions to maximize facial cleanliness and achieve environmental improvement for trachoma elimination: A review of the grey literature

**DOI:** 10.1371/journal.pntd.0006178

**Published:** 2018-01-25

**Authors:** Maryann G. Delea, Hiwote Solomon, Anthony W. Solomon, Matthew C. Freeman

**Affiliations:** 1 Department of Environmental Health, Rollins School of Public Health, Emory University, Atlanta, Georgia, United States of America; 2 Department of Disease Control, Faculty of Infectious & Tropical Diseases, London School of Hygiene & Tropical Medicine, London, United Kingdom; 3 Department of Control of Neglected Tropical Diseases, World Health Organization, Geneva, Switzerland; Mohammed Bin Rashid University of Medicine and Health Sciences, UNITED ARAB EMIRATES

## Abstract

**Background:**

Efforts are underway to scale-up the facial cleanliness and environmental improvement (F&E) components of the World Health Organization’s SAFE strategy for elimination of trachoma as a public health problem. Improving understanding of the F&E intervention landscape could inform advancements prior to scale-up, and lead to more effective and sustained behavior change.

**Methods/findings:**

We systematically searched for relevant grey literature published from January 1965 through August 2016. Publications were eligible for review if they described interventions addressing F&E in the context of trachoma elimination programs. Subsequent to screening, we mapped attributes of F&E interventions. We then employed three behavior change frameworks to synthesize mapped data and identify potential intervention gaps. We identified 27 documents meeting inclusion criteria. With the exception of some recent programming, F&E interventions have largely focused on intermediate and distal antecedents of behavior change. Evidence from our analyses suggests many interventions are not designed to address documented determinants of improved F&E practices. No reviewed documents endorsed inclusion of intervention components related to behavioral maintenance or resilience–factors critical for sustaining improved behaviors.

**Conclusions:**

If left unaddressed, identified gaps in intervention content may continue to challenge uptake and sustainability of improved F&E behaviors. Stakeholders designing and implementing trachoma elimination programs should review their F&E intervention content and delivery approaches with an eye toward improvement, including better alignment with established behavior change theories and empirical evidence. Implementation should move beyond information dissemination, and appropriately employ a variety of behavior change techniques to address more proximal influencers of change.

## Author summary

Trachoma is the world’s leading infectious cause of blindness. In light of a 2020 global target, the international trachoma community is intensifying its efforts to scale-up facial cleanliness and environmental improvement (F&E) components of the WHO-endorsed SAFE strategy for the elimination of trachoma as a public health problem. This is therefore an opportune moment to examine intervention content and delivery, and to consider their optimization. F&E interventions seek to change behaviors over the long term. Behavioral change and maintenance are predicated on behavioral antecedents, or precursors of change, and other determinants that may fall along different levels of influence. From the behavioral sciences literature, we know interventions are likely to be more effective if they target contextually-specific antecedents and causal determinants of behavioral change and maintenance. Evidence also suggests it is important to incorporate intervention techniques that are designed to address proximal change factors. However, F&E programming has largely focused on information dissemination via health and hygiene education, provision of water and sanitation facilities, and other interventions designed to address intermediate and distal behavioral factors. Intervention mapping and incorporation of techniques designed to address more proximal factors of behavioral change and maintenance have been deficient from F&E programming. Our review provides information regarding gaps in F&E intervention content and delivery that relevant stakeholders can use to understand why some interventions have not worked as intended, and strengthen F&E-related programming for trachoma elimination.

## Introduction

Trachoma, the world’s leading infectious cause of blindness [1], is a condition of the eye resulting from recurrent [2] ocular infection with certain serovars [3] of *Chlamydia trachomatis*. Infection with *C*. *trachomatis* can cause inflammatory changes, including conjunctivitis or pannus formation (i.e., abnormal growth of blood vessels over the cornea). Amongst some residents of endemic communities, conjunctival scarring [4], deposited as infections resolve, ultimately leads to trachomatous trichiasis and visual impairment or blindness. Globally, 3.2 million people suffer from trichiasis and are at risk of developing blindness, and an estimated 190 million people live in trachoma-endemic areas [5].

The World Health Organization endorses a multi-faceted approach for the elimination of trachoma as a public health problem. This approach is known as the SAFE strategy–**S**urgery for trichiasis, **A**ntibiotics to clear infection, and **F**acial cleanliness and **E**nvironmental improvement for reduced transmission of *C*. *trachomatis* [6]. Mass administration of antibiotics has proved effective at reducing the prevalence of active trachoma and ocular *C*. *trachomatis* infection in some populations [7, 8]. However, further development and incorporation of non-chemotherapeutic preventive interventions is likely needed to strengthen trachoma control, facilitate elimination of trachoma as a public health problem, and prevent recrudescence [9, 10].

Facial cleanliness and environmental improvement (F&E) efforts largely remain inconsistently operationalized and measured, in part because of a lack of clear, best-quality evidence regarding effective interventions [11, 12]. An improved understanding of F&E intervention content and delivery could facilitate cautious scale-up of appropriate F&E interventions. Understanding whether and to what extent these interventions are designed and effectively implemented to address key mechanisms of behavior change and maintenance can help guide refinement of F&E programming.

### Behavior change, facial cleanliness, and environmental improvement

Behavior change reflects a process of modification and transformation. This process is complicated by an assortment of change antecedents, which are precursors that need to be addressed before behavior change and maintenance can occur. Antecedents include factors such as perceived risk of infection or disease, attitudes and normative beliefs regarding improved practices, perceived and actual abilities to perform improved practices, self-regulation, and intentions to initiate and maintain the adoption of improved practices [13, 14]. Along with antecedents, other behavioral determinants (e.g., barriers and facilitators) mediate the adoption and translation of improved behaviors into action. In other words, in addition to potentially impeding initial behavioral uptake, these factors may also prevent the actual manifestation of improved behaviors [15]. For instance, one can possess improved behaviors, but due to unfavorable environmental conditions (e.g., contextual or technological barriers), be unable to translate those behaviors into the execution of improved practices. The complexity of behavior change is often reflected by the difficulty in achieving it; it requires more than simply raising awareness that change might be beneficial [16].

Although numerous behavior change intervention techniques exist, some are more successful at eliciting change than others. According to the *Theory of Triadic Influence*, various tiers of influence exist, and account for factors that have direct and indirect effects on behavior and practice [17]. See *S1 Appendix. Supplemental material* and S1 Fig for further information regarding this theory. Some intervention techniques operate at a more distal level of influence, and are therefore less likely to directly facilitate change when implemented in isolation. Others operate at a proximal level of influence, and are more likely to bring about change [17].

Critical evaluation of intervention attributes is necessary for understanding whether F&E content and delivery attend to key behavior change antecedents at proximal levels of influence. Throughout this paper, we distinguish between F&E content and delivery. Ensuring both intervention attributes are behavior-centered is critical to eliciting and maintaining improved behaviors and practices.

### Review rationale

Data from cross-sectional investigations suggest associations between improved F&E-related practices; more favorable water, sanitation, and hygiene (WASH) conditions; and lower prevalence of active trachoma [18–22]. Yet, few data from rigorous, experimental studies demonstrate the impact of specific F&E interventions on active trachoma and *C*. *trachomatis* infection [11, 12, 23]. It is crucial to consider the nature and fidelity of the interventions that generated effect estimates when interpreting evidence on the possible effectiveness of F&E in general. It is also important to understand the intervention landscape and potential gaps therein, to inform program improvement prior to scale-up.

Syntheses of the F&E literature have yet to fully describe approaches the trachoma community has taken to design and implement F&E interventions, and whether and to what extent they address important behavioral change and maintenance factors. Previous reviews have only examined data from peer-reviewed literature [11, 12, 21–23]. No known review has examined information from the grey literature, where many implementing organizations publish information regarding F&E content, delivery, and related metrics. Existing reviews have not collated information on frameworks, theories, and metrics the trachoma community has used to assess behavioral change elicited by F&E interventions. There is utility in thoroughly documenting details related to F&E content and delivery so as to:

1. Further clarify the types of F&E interventions that have produced the existing evidence base;
2. Identify opportunities for program improvements, including intervention design, planning, implementation, and monitoring; and
3. Inform evaluations of F&E programming to identify successful mechanisms of change.

Collating, synthesizing, and broadly disseminating information regarding F&E implementation from the grey literature can add to the existing understanding of the intervention landscape. More nuanced information regarding intervention content and delivery outlined in these materials is often not included in peer-review journal articles. These details can be used to identify programmatic gaps.

### Review objectives and research questions

The purpose of this review was to synthesize information regarding F&E intervention attributes to more fully describe the intervention landscape, so as to inform decision-making regarding policies, program implementation and evaluation, and possibly future research. We sought to determine which behavior change factors are addressed through F&E-related interventions endorsed in the grey literature. Our research questions were:

1. What are the implementation practices for F&E interventions employed in the context of trachoma elimination programs? What are the characteristics (i.e., content, delivery approaches) of interventions intended to address F&E? To what extent do current F&E interventions address known behavioral determinants (i.e., barriers and facilitators) mediating adoption of improved F&E behaviors?
2. Are trachoma programmers/researchers assessing behavioral change elicited by F&E interventions? Are trachoma programmers/researchers designing F&E interventions to address key behavior change factors, and which intervention techniques are being implemented to address these factors? Which behavioral frameworks are being used as the basis for designing F&E content and delivery approaches?

## Methods

We carried out a phased approach for establishing our search strategy and inclusion criteria, and collating and synthesizing information from the F&E-related grey literature. We circulated a draft review protocol to F&E subject matter experts, requesting feedback, which we used for further protocol refinement. We did not submit the protocol for publication in a peer-reviewed journal.

### Search strategy and eligibility criteria

We employed the PICOT framework [24], an extension of the traditional PICO framework [25], to construct our search strategy and guide decision-making regarding eligibility, data extraction, and analyses. PICOT is designed to improve precision of search results through articulation of details related to **P**articipants, **I**nterventions, **C**omparators, **O**utcome measures, and **T**ypes of eligible literature/study designs.

#### Search strategy

We used three specific information sources, as described below, to identify F&E-related grey literature. We conducted searches in English, Spanish, Portuguese, and French. However, we attempted to identify all relevant literature, regardless of publication language and location (i.e., we included literature published outside of peer-reviewed journals), produced between January 1965 and August 2016.

Information source 1. Electronic database searches: We used a set of Boolean search terms (S1 Table) to search *Open Grey* and *New York Academy of Medicine Grey Literature Report* databases.

Information source 2. Systematic review of key websites: We performed a standardized search, using pre-determined keywords (S2 Table), to identify relevant literature published on or housed within the websites of key organizations implementing trachoma programming or conducting related operational or academic research. Key organizations included those participating in the International Coalition for Trachoma Control (ICTC) and WHO Collaborating Centers for Trachoma (WHO-CCT). See Supplemental Material for a full list of the member organizations targeted for this search. In addition to these network websites, reviewers systematically searched the websites of each partner or affiliate participating in these networks.

Information source 3. Literature solicitation from implementers and researchers: Reviewers approached individuals working in the trachoma sector, commencing with members of staff of WHO-CCT and ICTC member organizations, requesting that they submit relevant literature for review. We engaged these stakeholders to identify documents that were currently in process or previously unidentified through our searches of other information sources (see Supplemental Material for a list of member organizations engaged via ICTC and WHO-CCT). We completed the process by emailing the ICTC listserv twice, and through personal communication during a meeting of WHO-CCT.

#### Screening and literature selection

Once F&E grey literature documents were identified, we screened them to determine which were eligible for inclusion in our review, as per our PICOT inclusion and exclusion criteria.

Participants: We considered literature referencing F&E interventions and outcomes implemented and detected amongst children and adults. Settings included areas that were endemic for trachoma (hyper-, meso-, or hypo-endemic for trachoma), and in which trachoma elimination programs were operating at the time of publication. Our operational definition of trachoma elimination program aligned with WHO’s *Guide to Trachoma Control Programs for the Prevention of Blindness* [26], which specifies that programs to control trachoma should include the following elements:

1. Assessment of the problem and establishment of priorities;
2. Allocation of resources;
3. Chemotherapeutic intervention;
4. Surgical intervention to correct lid deformities;
5. Training and utilization of local health aids and other non-specialized health workers;
6. Health education and community participation; and
7. Evaluation of intervention programs.

Interventions: The interventions of interest for this review were broad, yet reflect those addressing facial cleanliness and environmental improvement in the context of trachoma elimination programs, including but not limited to WASH interventions. Literature describing interventions aimed at improving at least one of the following were eligible for inclusion in the review:

- Facial cleanliness;
- Personal hygiene more generally (e.g., hygiene/health education, promotion of frequent bathing, promotion of handwashing, construction of washing facilities, promotion of clothes washing or activities to reduce transmission by other potential fomites);
- Environmental improvement, such as (see S1 Appendix for further details):
  ○ Provision of water supply, under the auspices of trachoma elimination programs (i.e., standalone water schemes were not eligible), to support the facilitation of facewashing, facial cleanliness, handwashing, and personal hygiene, more broadly;
  ○ Control of *Musca sorbens* populations, including:
    ■ Safe human feces disposal (e.g., interventions aimed at improving latrine coverage [provision of sanitation hardware: latrines, child potties] and utilization [progression along the sanitation ladder, cessation of open defection through demand-side or other sanitation programming, or improved sanitation services]), including feces disposal for infants and young children as well as disabled and elderly individuals;
    ■ Insecticide spraying;
  ○ Reduced crowding (e.g., limiting crowding in sleeping quarters).

We reviewed documentation of F&E-related interventions from grey literature that endorsed the interventions either in isolation or in combination with other interventions.

Comparators: We did not limit inclusion to literature that made comparisons between specific interventions and counterfactuals (e.g., alternative interventions or no interventions).

Outcome measures: Grey literature describing interventions aimed at modifying at least one of the following F&E outcomes (and behavioral proxies thereof) were eligible for inclusion in the review:

- Facial cleanliness outcomes or proxies thereof (e.g., researcher-determined ‘clean face’; absence of ocular discharge, nasal discharge, flies on face; fly-eye contacts; ‘any dirt’ on face; proportion of collected water allocated to facewashing; reported or observed facewashing; reported or observed facewashing practices [e.g., use of soap and/or towel, wiping practices]). Some evidence suggests that such measures have low inter- and intra-rate reliability [27]. However, these metrics have been used in trachoma elimination programs, and in the absence of more reliable measures, these outcomes were incorporated in order to include interventions addressing proxy measures related to facial cleanliness;
- Environmental improvement outcomes (e.g., water availability [e.g., access to and sustainability of water supply; distance to water source, often indicated by time to source or collection time; amount of water collected; proportion of collected water allocated to personal hygiene, handwashing], latrine coverage, latrine utilisation, prevalence of open defecation, fly densities);
- Hygiene outcomes (e.g., bathing practices, washing practices related to potential fomites, handwashing practices);
- F&E-specific behavioral change outcomes or proxies thereof, including:
  ○ Changes in perceived risk/vulnerability and severity (including consequences of disease/infection, causal determinants of ill/good health), knowledge (including health/hygiene knowledge–disease transmission and prevention, action knowledge), attitudes (cost and benefit beliefs, affective feelings), beliefs regarding improved practices related to any of the following: facewashing, personal hygiene (bathing, handwashing), sanitation (including adoption of technologies), crowding, and animal husbandry
  ○ Changes in availability of resources and/or skills required to perform improved F&E practices (e.g., action capacity)
  ○ Changes in F&E-related norms (i.e., social and non-social normative and non-normative beliefs), including [28, 29]:
    ■ Empirical expectations: (i.e., descriptive norms) social, non-normative beliefs about what other people actually do;
    ■ Normative expectations: (i.e., injunctive or “social norms”) social, normative beliefs about what other people think one ought to do (or ought not do);
    ■ Factual beliefs: (i.e., collective habits) non-social, non-normative beliefs about the world; and
    ■ Moral norms: (i.e., personal [i.e., non-social] normative beliefs) personal perceptions about what ought to be done (or what ought not be done)
  ○ Changes in confidence in performance of improved F&E-related practices at the individual (i.e., self-efficacy) and community (i.e., collective efficacy) levels [30]
  ○ Individual- or household-level characteristics associated with adoption of improved F&E behaviors
  ○ Outcome or process evaluations of interventions that include behavioral outcomes such as adoption of improved F&E practices.

Types of eligible literature: Grey literature, including technical guidelines and resources, implementer reports and exchanges, formative research reports, program documents, policy and advocacy pieces, progress reports, and white papers were eligible for inclusion.

#### Exclusion criteria

Literature was excluded if it was: 1) not published in targeted grey literature databases or on targeted websites, as outlined above, and 2) not provided to the authors via the literature solicitation from ICTC and WHO-CCT member organizations. Routine surveillance reports were not eligible. Papers that were published as conference abstracts or proceedings were also excluded from the review, as the data presented in these types of publications are subject to change when published in peer-reviewed journals and/or as final programmatic reports [31].

### Data extraction and collation: Mapping of endorsed F&E-related intervention content

We performed a mapping exercise [32] to broadly survey the nature of F&E content and delivery, and reported results regarding lessons learnt and promising practices. The purpose of this mapping phase was to extract data that could be synthesized during the subsequent analysis phase. One reviewer employed a semi-structured instrument to rapidly, yet systematically survey the content of all documents included in the review. A second reviewer employed the same instrument to complete a validity check of 10% of the reviewed literature. When inconsistencies arose, the two discussed the results, and came to a consensus.

In addition to information related to document identification and publication, we mapped five categories of characteristics via our semi-structured data extraction instrument (*S1 Appendix*).

### Synthesis of results

Due to anticipated heterogeneity in methodology and reporting styles, we employed various, complementary qualitative methods for data synthesis. Based on the type of information provided in the F&E grey literature, we determined that thematic and narrative synthesis approaches were appropriate methods for the purposes of this review [33].

Our synthesis activities focused on categorizing intervention activities, cataloguing documented behavioral determinants of improved F&E adoption, and determining the types of behavioral antecedents and intervention techniques addressed through interventions endorsed in the grey literature. We used these synthesis activities, described in further detail below, to answer our review questions. Each activity resulted in a unique analytical output. Cumulatively, the syntheses generated a descriptive overview of F&E content and delivery endorsed in the grey literature, and pinpointed gaps in the intervention landscape.

#### Synthesis activity 1: Thematic analysis of endorsed F&E-related intervention activities

To categorize intervention activities and identify emerging themes related to interventions promoted in the grey literature, we performed an applied thematic synthesis [34]. First, we catalogued all intervention activities endorsed in documents selected for inclusion in the review. Once we listed all intervention activities, we identified emergent intervention themes (i.e., categories of intervention activities). We then developed a matrix in which each column represented a single included document, and each row represented one emergent intervention theme. In order to visualize the results of our thematic analysis, we transformed the matrix into a cluster heat map [35] with each cell representing whether, and to what extent the respective literature endorsed the theme in question. We used a stop-light color system to indicate whether interventions described in the reviewed literature explicitly endorsed the emergent F&E intervention categories (green cells), implicitly endorsed the intervention categories (yellow cells), or did not mention the intervention categories (red cells). Rows of intervention themes were placed in order, according to their level of influence [17], with those least influential in changing behavior (i.e., ultimate/distal influencers) situated at the top of the matrix, and those most influential in changing behavior (i.e., proximal influencers) situated at the bottom.

#### Synthesis activity 2: Synthesis of documented behavior change determinants (i.e., barriers and facilitators) mediating the adoption of improved F&E-related practices

Determinants of improved F&E practices are context-specific; however, many barriers and facilitators transcend contexts. We used the *Integrated Behavioral Model for Water*, *Sanitation*, *and Hygiene* (IBM-WASH) framework [36] to synthesize information regarding behavior change determinants that were indicated as known mediators of F&E practices. While we did not track information against the various hierarchical dimensions of the IBM-WASH framework, we did organize barriers and facilitators into the framework’s three overarching dimensions: contextual, psychosocial, and technological determinants.

#### Synthesis activity 3: Keyword exercise identifying behavioral factors and antecedents addressed through endorsed F&E-related intervention approaches

Further to the categorization of intervention activities, we classified the types of intervention techniques endorsed as well as the behavior change factors addressed through these techniques. In order to do so, we used the *Risks*, *Attitudes*, *Norms*, *Abilities*, *and Self-regulation* [RANAS] *approach to systematic behavior change* [13]. This framework is grounded in theories of change, explores a variety of behavior change antecedents, or change mechanisms, and tracks them against specific categories of intervention techniques. By developing keywords that aligned with our adaptation of the RANAS framework’s behavioral factors and related domains and sub-domains, we tracked the attributes of F&E-related intervention components against the RANAS framework. We slightly adapted the framework by introducing the creation of enabling environments, and shifting behavioral reinforcement from the infrastructural and ability category to the planning and relapse prevention category, as we postulate positive reinforcement can help with action planning and prevent relapse. Subsequent to keyword creation, we searched each included document for targeted keywords, and recorded whether the behavior change factors and techniques indicated were explicitly, implicitly, or not at all endorsed. Although authors of the grey literature may not have explicitly mentioned some behavior change dimensions, we interpreted and/or inferred whether the F&E-related interventions described did, indeed, attend to certain behavior change factors related to the RANAS framework. We used this approach because we recognized that those designing and implementing trachoma elimination programs may not be using the RANAS or other behavior change frameworks as foundations to guide their interventions. Therefore, they would not necessarily use related terminology when describing intervention content and delivery. For ease of tracking the total number of documents describing F&E-related interventions that touched on the respective behavioral factors and accompanying change mechanisms, we tabulated explicit and implicit results (S3 Table). Also see S3 Table for the operational definition of each dimension that taps to the five RANAS domains.

In order to elucidate the conceptual and behavioral frameworks the trachoma community is using to inform the design of its F&E interventions, we catalogued this information in a table (S4 Table).

## Results

**Fig 1:**
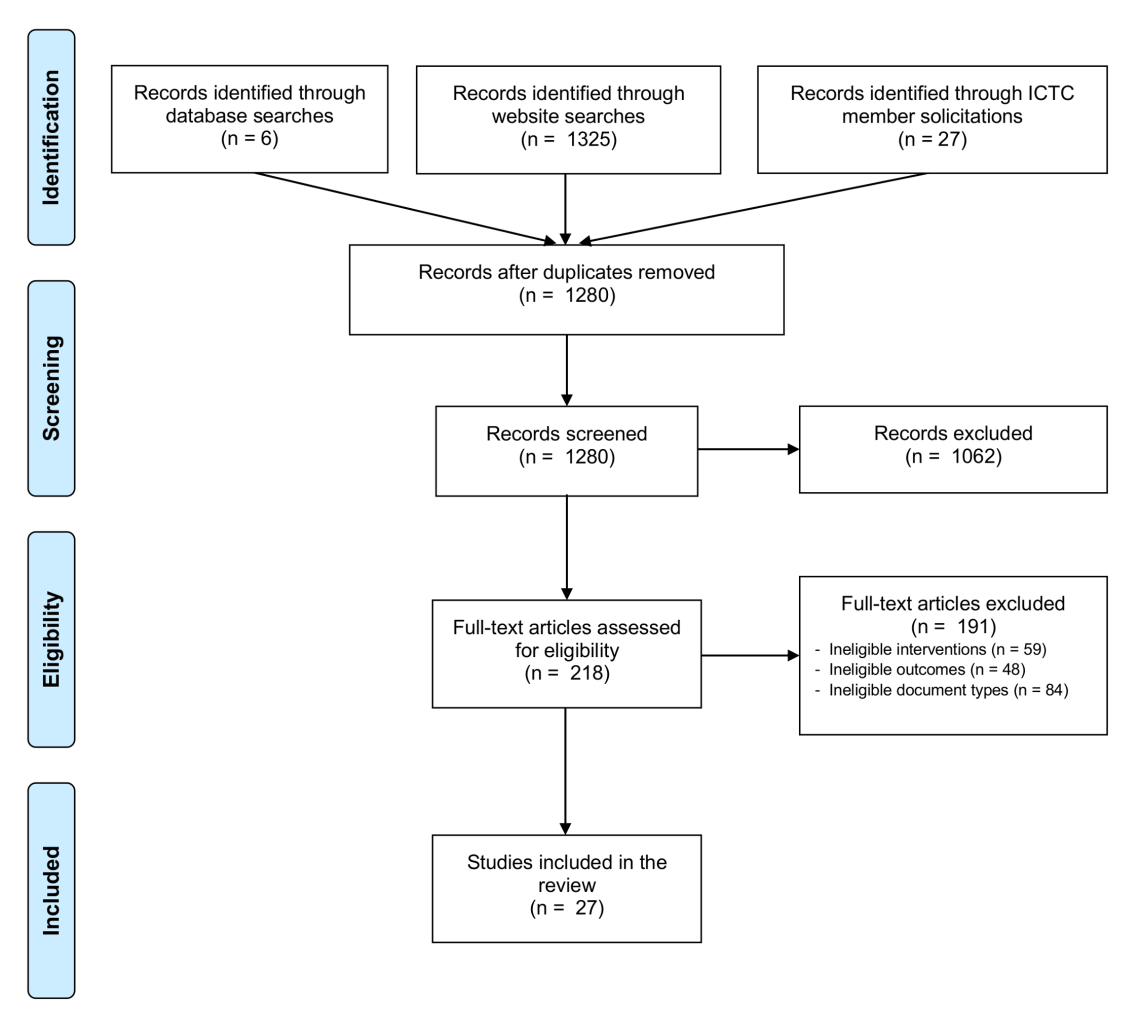
PRISMA diagram of publication flow.

Following screening and eligibility assessment, we identified 27 documents that met inclusion criteria (Fig 1).

### Summary of reviewed grey literature

Table 1 lists the documents included, and provides a summary of publication information as well as the means by which we identified the document, and the type of content contained therein. The grey literature was produced by a range of trachoma and NTD networks/coalitions, non-governmental development organizations, multilateral and technical organizations, research institutions, and government partners. Several documents were produced by a combination of different stakeholder groups. The vast majority of included literature (96%, n = 26) was identified via keyword searches on target websites. Amongst those, nearly half (42%, n = 11) of the documents were also detected via another mode of identification. Only one document was not first identified through our web search. This was the *Handbook on Community-Led Total Sanitation* (CLTS) [37], which we confirmed was identified via our web search, but was initially screened out, as it met a pre-defined exclusion criterion (i.e., it represented a general WASH-related resource that did not specifically touch on inclusion of CLTS in trachoma elimination programs). We later included this resource in the review because several practitioners identified it as a key resource for trachoma programming during the literature solicitation process. Over half of the documents included (55%, n = 15) represent technical guidance and resource documents. Six (22%) are implementer reports, and the remaining literature represented a combination of policy/advocacy pieces and other documents.

**Table 1:** Summary of reviewed F&E-related grey literature.

Title	Organization(s)	Date of publication	Means of identification	Type of document
***F&E/SAFE-specific grey literature captured via search***				
Health promotion partnerships for trachoma elimination [38]	Indigenous Eye Health Unit, University of Melbourne	September 2015	W, I	**Implementer report/exchange; policy/advocacy piece** intended to provide information on a spectrum of health promotion strategies for engagement, advocacy, and the elimination of trachoma in Australia's Northern Territories
All you need for F&E: A practical guide for planning and partnering [39]	ICTC	August 2015	W, I	**Technical guidance** for design, implementation, monitoring and evaluation of F&E interventions
Trachoma Action Planning: A planning guide for the national elimination of blinding trachoma [40]	ICTC, KCCO	June 2015	W	**Technical guidance** for developing effective and efficient trachoma action plans that attend to the entire SAFE strategy
Education and trachoma [41]	School of Population and Global Health, University of Melbourne	April 2015	W, I	**Implementer report/exchange** regarding important linkages between trachoma elimination & education
Formative research assessment and guidelines: Facial cleanliness and environmental sanitation [42]	JHU—CCP	August 2014	W	**Technical guidance** for implementation of F&E-related formative research
Protocol and methods for trachoma situation analysis: Using a systematic process for understanding F&E for trachoma programs [43]	JHU—CCP, Sightsavers, ICTC	February 2014	W	**Technical guidance** for performing an F&E-centric trachoma situational analysis
Understanding individual and contextual factors for development of a behavior change communication campaign for trachoma prevention in Busoga and Karamoja Regions, Uganda [44]	Sightsavers, JHU—CCP	January 2014	W	**Implementer report** of findings from formative research intended to guide the development of an F&E behavior change communication campaign
ICTC principles for F&E [45]	ICTC	2014	W	**Technical resource** to inform programming and partnerships for F&E intervention implementation
Research to inform the development of behavior change interventions for "F" and "E" of the SAFE strategy in Turkana and Marsabit, Kenya [46]	LSHTM, Kenya MoH	July 2013	W	**Implementer report o**f findings from formative research intended to guide development of behavior change interventions for F&E
WASHing away blinding trachoma [47]	Sightsavers, WaterAid	April 2013	W	**Policy/advocacy piece** for the comprehensive implementation of the SAFE strategy
Clean faces, strong eyes [48]	Indigenous Eye Health Unit, University of Melbourne	May 2012	W, I	**Implementer report/exchange** intended to provide information regarding experiences with improving F-related knowledge, attitudes, and practices via health promotion & social marketing
How communities can control for trachoma without a big budget [49]	ITI, The Carter Center	2012	W, D	**Technical guidance; policy/advocacy piece** for tangible, low-/no-cost options for community engagement in trachoma control
The end in sight: 2020 INSight [50]	ICTC	July 2011	W	**Technical guidance; policy/advocacy piece** for global strategic planning and guidance for national level planning and implementation of the SAFE strategy
Trachoma resource book [51]	Indigenous Eye Health Unit, University of Melbourne	May 2010	W, I	**Technical guide** of resource materials for practical support for the implementation of trachoma control activities in Australia
Women and trachoma: Achieving gender equity in the implementation of SAFE [52]	KCCO, TCC, The Elfenworks Foundation	February 2009	W	**Technical guidance** for gender-sensitive implementation of SAFE strategy, with specific chapters on behavior change and achieving equity in F&E implementation
The 'ngisipet' and trachoma prevention: solving the latrine problem in nomadic tribes [53]	Ol Malo eye Project, Ol Malo Trust	December 2007	W, D	**Implementer report/exchange** regarding a non-facility-based sanitation intervention
Communicable Disease Network Australia (CDNA) national guidelines for the public health management of trachoma [54]	Communicable Disease Network Australia, Australia Health & Protection Principal Committee	March 2006	W, I	**Technical guidance** for the implementation & monitoring of the national trachoma control program in Australia
Implementing the SAFE strategy for trachoma control: A toolbox of interventions for promoting facial cleanliness and environmental improvement [55]	The Carter Center, ITI	January 2006	W, I	**Technical guidance** for the implementation of interventions promoting F&E
Pit latrines for all households: The experience of Hulet Eju Enessie Woreda, Amhara National Regional State, Northwest Ethiopia [56]	The Carter Center, Ethiopian Federal Ministry of Health, Amhara Regional Health Bureau	September 2005	W	**Implementer report/exchange** intended to provide information on the experience of improving latrine coverage in Hulet Eju Enessie *woreda*, share lessons learnt & best practices
The SAFE strategy: Preventing trachoma—A guide for environmental sanitation and improved hygiene [57]	WHO, ITI	2000	W, D	**Technical guidance** for environmental sanitation & improved hygiene for trachoma prevention
Teaching series No. 07 –Trachoma [58]	International Center for Eye Health, LSHTM	1999	W	**Technical resource—teaching slides** on trachoma & the SAFE strategy
Trachoma: A women's health issue [59]	The Global Alliance for Women's Health, Edna McConnell Clark Foundation	1997	W	**Policy/advocacy piece** highlighting trachoma as a women's health issue
Achieving community support for trachoma control: a guide for district health work [60]	WHO	1995	W, D	**Technical guidance** for district health workers to cultivate community support for & encourage community action toward trachoma control initiatives—full SAFE strategy covered
***General WASH-NTD grey literature captured via search***				
Water, sanitation & hygiene for accelerating and sustaining progress on neglected tropical diseases: A global strategy 2015–2020 [61]	WHO	August 2015	W	**Technical guidance** for joint cross-sectoral/program planning, implementation, and evaluation of activities to achieve common goals related to WASH and NTD initiatives
WASH and the neglected tropical diseases: A global manual for WASH implementers [62]	Sightsavers, DFID, ITI, Children Without Worms, WaterAid, WASH Advocates, Emory University, CARE USA	February 2014	W	**Technical guidance** for WASH implementers to address five key NTDs through collaborative monitoring of NTD-specific health outcomes & impacts to inform program & policy change
WASH: The silent weapon against NTDs [63]	WaterAid, NNN	2012	W	**Policy/advocacy piece** for better WASH-NTD integration at global to community levels
***Other grey literature captured***				
Handbook on community-led total sanitation* [37]	Institute for Development Studies, Plan UK	March 2008	I	**Technical guidance** for the implementation of community-led total sanitation (a demand-side sanitation intervention)

NOTES: Literature is presented by date of publication

* Document provided by multiple ICTC member organizations during literature solicitation process, and implemented in the context of trachoma elimination programs, therefore included in the review

Organizational acronyms: ICTC = International Coalition for Trachoma Control; KCCO = Kilimanjaro Center for Community Ophthalmology; JHU—CCP = Johns Hopkins University—Center for Communication Programs; LSHTM = London School of Hygiene & Tropical Medicine; ITI = International Trachoma Initiative; DFID = Department for International Development; WHO = World Health Organization; NNN = Neglected Tropical Diseases NGDO Network

Means of identification: I = ICTC member literature solicitation; W = targeted website key word search; D = database search

Table 2 outlines types of interventions, content and delivery approaches, and level of implementation. Twenty-three (85%) of the documents contained F&E-specific content, while three (11%) more generally described content and delivery along the WASH-NTD nexus. The authors are aware that several funded trachoma elimination programs are using participatory hygiene and sanitation transformation (PHAST) and children’s hygiene and sanitation training (CHAST). However, no related resources were identified through our searches or literature solicitation process. As such, no other general WASH resources were deemed appropriate for inclusion in the review. Several of the documents included addressed the creation of enabling environments via cross-sectoral partnerships and collaboration, national and sub-national polices and guidelines, and community-level contracts and by-laws. Other documents focused largely on local, community, school, and individual-level intervention activities and implementation approaches. The literature covered a mix of intervention types, from F&E advocacy, design, planning, and implementation to monitoring and evaluation, collation, and synthesis of resulting information.

**Table 2:** Summary of implementation details from the F&E grey literature.

Title	Intervention content and delivery	Level of implementation	Type of intervention
***F&E/SAFE-specific grey literature captured via search***			
Health promotion partnerships for trachoma elimination	Employing medical, behavioral, & socio-economic approaches via multi-faceted health education strategies to incorporate 2 key F&E-related messages (i.e., “clean faces, strong eyes” & “safe & functional bathrooms”) into programming at numerous entry points within the community setting (e.g., cultural events, schools, early child & family well-being settings, clinics)	National, sub-national, community (e.g., school, early child & family well-being settings, clinics) & individual	Design, planning, implementation
All you need for F&E: A practical guide for planning and partnering	Creating and fostering cross-sectoral partnerships and collaboration, developing and implementing F&E interventions that facilitate related and sustainable behavior change, planning and monitoring of F&E activities and outcomes, sustainability thereof	National, sub-national, community	Design, planning, implementation
Trachoma Action Planning: A planning guide for the national elimination of blinding trachoma	Action planning, including identification of F&E partners, completion of a full F&E situation analysis, cataloguing of existing hygiene and related behavior change communication programs/events	National, sub-national (e.g., district)	Planning
Education and trachoma	Integrating facewashing & improved hygiene promotion into school curricula; establishing clean faces as the new social norm; ensuring safe & functional washing facilities are present & properly maintained with access to water, soap, paper towels, tissues, mirrors, & bins	Sub-national, school, community	Planning, implementation
Formative research assessment and guidelines: Facial cleanliness and environmental sanitation	Collating national and sub-national (e.g., district) level data, generating qualitative & quantitative data at community, household, and individual levels	National, sub-national, community, individual	Data generation, collation, synthesis
Protocol and methods for trachoma situation analysis: Using a systematic process for understanding F&E for trachoma programs	Creating and fostering networks and collaboration, collation of national and sub-national (e.g., district) level data	National, sub-national (e.g., district)	Design, planning
Understanding individual and contextual factors for development of a behavior change communication campaign for trachoma prevention in Busoga and Karamoja Regions, Uganda	Utilizing mixed methodological formative research that provides information to inform development of an F&E BCC campaign	Community	Data generation, collation, synthesis, design, planning
ICTC principles for F&E	Designing and implementing community-centered programs and interventions via active engagement of affected communities in planning, decision-making, and relevant training, with explicit efforts to involve marginalized populations	National, sub-national, community, with focus on action at national to district levels	Designing, planning, implementation (including M&E)
Research to inform the development of behavior change interventions for "F" and "E" of the SAFE strategy in Turkana and Marsabit, Kenya	Utilizing mixed methodological formative research that provides information to inform development of behavior change interventions for F&E	Community	Data generation, collation/synthesis, design, planning
WASHing away blinding trachoma	Administering a holistic approach to the implementation of the SAFE strategy that ensures the integration of F&E components from the outset of trachoma elimination and control programs to ensure the underlying causes, as well as the symptoms of the disease are addressed to break the transmission cycle	Global, national, sub-national, community	Design, implementation, advocacy
Clean faces, strong eyes	Developing and employing culturally appropriate health promotion resources and social marketing strategies via a consultative process to ensure: 1) the indigenous culture is respected; 2) clear & holistic messages; and 3) local people, environment, and popular culture are involved	National, sub-national, community	Design, planning, implementation
How communities can control for trachoma without a big budget	Designing and implementing community-based interventions that include promotion and uptake of individual, household, and community behaviors that prioritize and act to ensure faces are clean, household members (all community members) dispose of their feces in a safe manner, and households and communities are free of materials that attract flies	Community, household, individual	Design, planning, implementation
The end in sight: 2020 INSight	Employing five guiding principles that provide a framework for the path to elimination	Global, national, sub-national (e.g., district)	Planning, implementation
Trachoma resource book	Administering a program development, implementation, & evaluation approach that involves engagement and partnership with local community at all levels, and includes genuine collaboration & consultation, ensuring comprehensive implementation of the SAFE strategy via a workforce with appropriate knowledge, skills, & experience in trachoma control through regular training & support	National, sub-national, community	Design, implementation, monitoring
Women and trachoma: Achieving gender equity in the implementation of SAFE	Utilizing a gender-sensitive program approach via the use of strategies to: 1) recognize gender-specific attitudes toward & motivators of improved behaviors, 2) determine existing positive behaviors and effective channels of communication within the community—noting similarities & differences between men/women, and 3) segment audience to facilitate concurrent targeting of different groups	Community	Design, planning, implementation
The 'ngisipet' and trachoma prevention: solving the latrine problem in nomadic tribes	Engaging the community for the development of a locally acceptable and culturally appropriate alternative to improving environmental conditions amongst nomadic tribes	Community	Design, implementation
CDNA national guidelines for the public health management of trachoma	Ensuring engagement with local communities when planning, implementing, and evaluating trachoma programs; creating & fostering collaboration between public health units, primary health care, and other services working towards trachoma elimination; collection of high quality data to monitor & evaluate progress towards trachoma elimination by improving coverage, completeness, and timeliness of surveillance data in accordance with the minimum national trachoma dataset	Reporting & coordination: national; planning, implementation, monitoring: sub-national, community	Planning, implementation, monitoring
Implementing the SAFE strategy for trachoma control: A toolbox of interventions for promoting facial cleanliness and environmental improvement	Employing *Hygiene Improvement Framework* that addresses three areas to reduce transmission: 1) hygiene promotion—encouraging existing practices (e.g., face- & handwashing) and new practices (e.g., individual towel use, safe child feces disposal); 2) access to & utilization of household & community hardware (e.g., HH latrines, community water & waste management systems); and 3) enabling environments	Community	Design, implementation, monitoring & evaluation
Pit latrines for all households: The experience of Hulet Eju Enessie Woreda, Amhara National Regional State, Northwest Ethiopia	Mobilizing community (e.g., gaining community acceptance & support via triggering of latent demand/demand creation for sanitation & hygiene facilities); leaders working through existing structures, with reference to community practices & cultural norms; conducting training-of-the-trainer to empower community leaders, early adopters, community to build latrines, for demonstration purposes to encourage participation; conducting educational sessions on the benefits of latrine ownership & use	Community, with support from sub-national & national	Design, planning, implementation
The SAFE strategy: Preventing trachoma—A guide for environmental sanitation & improved hygiene	Employing an approach for participatory selection of interventions intended to address environmental sanitation & improved hygiene for prevention	Community	Implementation
Teaching series No. 07 –Trachoma	Implementing trachoma elimination and control programs via the district healthcare system to ensure agreement with the public health framework; ensuring communities take part in trachoma control activities, with assistance of members to identify factors which encourage transmission	Sub-national, community	Design, implementation, monitoring
Trachoma: A women's health issue	Creating & fostering cross-sectoral collaboration to enhance investment in community-based interventions for trachoma prevention & control via a women's health lens	Global, national, community	Design, implementation, advocacy
Achieving community support for trachoma control: a guide for district health work	Administering a community-based, participatory approach to learn from the community what they are willing & interested in doing to prevent trachoma, identify how selected interventions can be integrated into existing village health, education, religious, and social activities	Community	Design, planning, implementation
***General WASH-NTD grey literature captured via search***			
Water, sanitation & hygiene for accelerating and sustaining progress on neglected tropical diseases: A global strategy 2015–2020	Creating and fostering cross-sectoral partnerships and collaboration	Global, national	Planning
WASH and the neglected tropical diseases: A global manual for WASH implementers	Integrating WASH-NTD initiatives, creating and fostering cross-sectoral collaboration via joint monitoring of trachoma-specific health outcomes and impacts to inform programmatic and policy change	National, sub-national, community	Design (of integrated programs), planning
WASH: The silent weapon against NTDs	Shifting program implementation to a *Service Delivery Approach* that incorporates behavior change components; creating & fostering cross-sectoral partnerships; designing/implementing demand-side or combined demand-side & supply-side interventions	Global, national, sub-national, community	Design, planning, implementation
***Other grey literature captured***			
Handbook on community-led total sanitation*	Employing a community-based, participatory approach; stimulating latent demand and/or demand creation; cooperative approach that focuses on the collective benefit of stopping open defecation by concentrating on changing behaviors of the whole community as opposed to individuals	Community	Design, planning, implementation

### Findings related to RQ1. Implementation practices in the context of trachoma programs

#### Characteristics of endorsed F&E-related intervention activities

**Fig 2:**
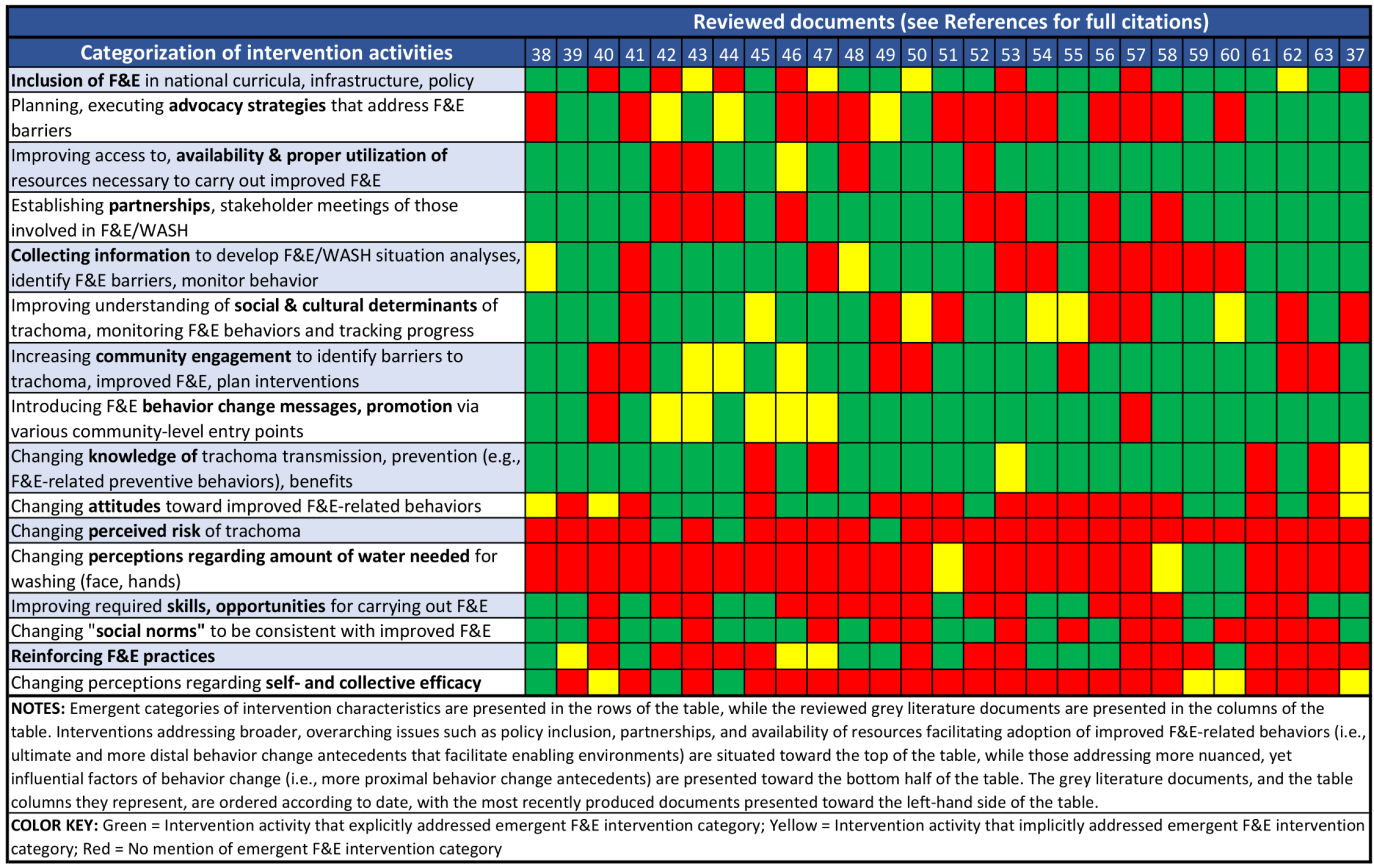
Heat map visualizing emergent themes related to F&E intervention activities.

The heat map (Fig 2) suggests that existing interventions address enabling environments, introduce behavior change messages and promotional activities (20 explicitly, 5 implicitly), and focus on improving knowledge (21 explicitly, 2 implicitly) regarding trachoma transmission and prevention. However, in much of the literature, such interventions are not coupled with activities that address more influential (i.e., proximal) behavior change antecedents known to facilitate successful and sustainable change. This is evidenced through the relatively high proportion of green cells in rows 1 to 9 compared to the relatively low proportion of green cells in rows 10 to 16. For example, many documents focused on introducing knowledge related to trachoma (row 9), but few also gave focus to perceptions regarding the amount of water required to wash one’s face and hands (row 12), or self- and collective efficacy (row 16). The intervention activities presented in rows 10 to 16 of the table refer to those intended to:

- improve attitudes toward improved F&E-related behaviors and the skills and opportunities required to carry them out;
- address perceptions regarding: risk of trachoma, self- and collective efficacy, the amount of water required to wash one’s face and hands;
- change normative beliefs (referred to generally in the grey literature as “social norms”), such that they are consistent with improved F&E practices; and
- reinforce improved F&E practices (e.g., modelling of or praising someone for improved practices, use of role models for promotion, recommending to others that they carry out improved practices).

#### Documented behavior change determinants mediating the adoption of improved F&E practices

Across the three IBM-WASH dimensions, psychosocial determinants received greatest focus when consideration was given to uptake of improved F&E practices (Table 3). The majority (76%, n = 22) of the 29 categories of cited behavioral determinants portray barriers of improved F&E adoption.

**Table 3:** Behavioral determinants of improved F&E practices presented in the grey literature.

**F&E-related barriers**
***Contextual barriers***
**Access to, availability and appropriate allocation of resources required** to carry out improved F&E-relate practices*
**Hydrogeological factors** posing challenges to installation of facilities (e.g., arid land, lack of water; or conversely, high water tables/water-logging)
***Psychosocial barriers***
**Social norms** (e.g., acceptance of "dirty face", "old" people having poor vision, defecating in the open accepted as social norms in many contexts)
**Lack of knowledge/awareness** regarding health/hygiene implications and/or benefits of improved F&E-related behaviors/practices
**Personal hygiene** (facewashing, handwashing, body washing) is **not a priority** with regard to **allocation of household water resources**
Household and community-level **decision-making authority** may pose challenges to acting on improved F&E-related behaviors
**Knowledge** about trachoma transmission and prevention **does not translate to related improved (i.e., preventive) F&E-related behaviors, practices**
Varying **beliefs about the causes of ill health**/trachoma (e.g., old age, evil eye, discussion of the disease means questioning the will of God)
**Perceptions** (e.g., those regarding improved behaviors, disease transmission, amount of water necessary for facewashing)
**Lack of skills** required to perform improved F&E-related practices and/or act on improved behaviors
**Perceived risk** of trachoma is low
**Poor attitudes** toward improved behaviors
Practicing improved F&E-related behaviors induces **shame, is taboo, or goes against social expectation**s (e.g., washing young children is culturally taboo)
**Ubiquitous presence of fece**s in environment **does not trigger feelings of disgust, or facilitate intrinsic motivation to remove them** from the environment
**Competing priorities/constraints on time** necessary to execute improved F&E-related practices
**Lack of motivation** to execute improved F&E-related practices
***Technological barriers***
**Coordination costs** related to facilities installation
Facilities **installation, operation & maintenance costs** and other issues with their lack of acceptability (e.g., promoted technologies not preferred, acceptable)
**Poor functionality (and safety)** of F&E/WASH facilities & management committees
People **lack the materials and skills necessary to install** household latrines
**Perceptions** that **latrines are not a valuable** use of time/resources
**Reliance on non-governmental development organizations** to install/repair/upgrade sanitation and hygiene facilities disincentivizes households to do so themselves
**F&E-related facilitators**
***Contextual facilitators***
**Advocacy to increase political will** and funding at both a global and national level, better coordination at global to community levels
**Enabling environments**: Global, national, sub-national policies and/or community by-laws/contracts
**Piggy-backing** F&E-related behaviors and practices onto existing (related) programs, using existing infrastructure
***Psychosocial facilitators***
**Habit formation** of improved F&E-related practices
**Positive reinforcement** for execution of improved practices (e.g., praise, awards, modelling)
Local and community promoters **feel comfortable** talking about/advising on hygiene
***Technological facilitators***
Employing demand-side or combined demand- and supply-side interventions to gain acceptability of improved facilities/use thereof

NOTE

* There are some psychosocial aspects of this determinant as well

### Findings related to RQ2. Behavior change assessment within F&E interventions

#### Behavioral factors, antecedents addressed through endorsed F&E-related intervention approaches

**Fig 3:**
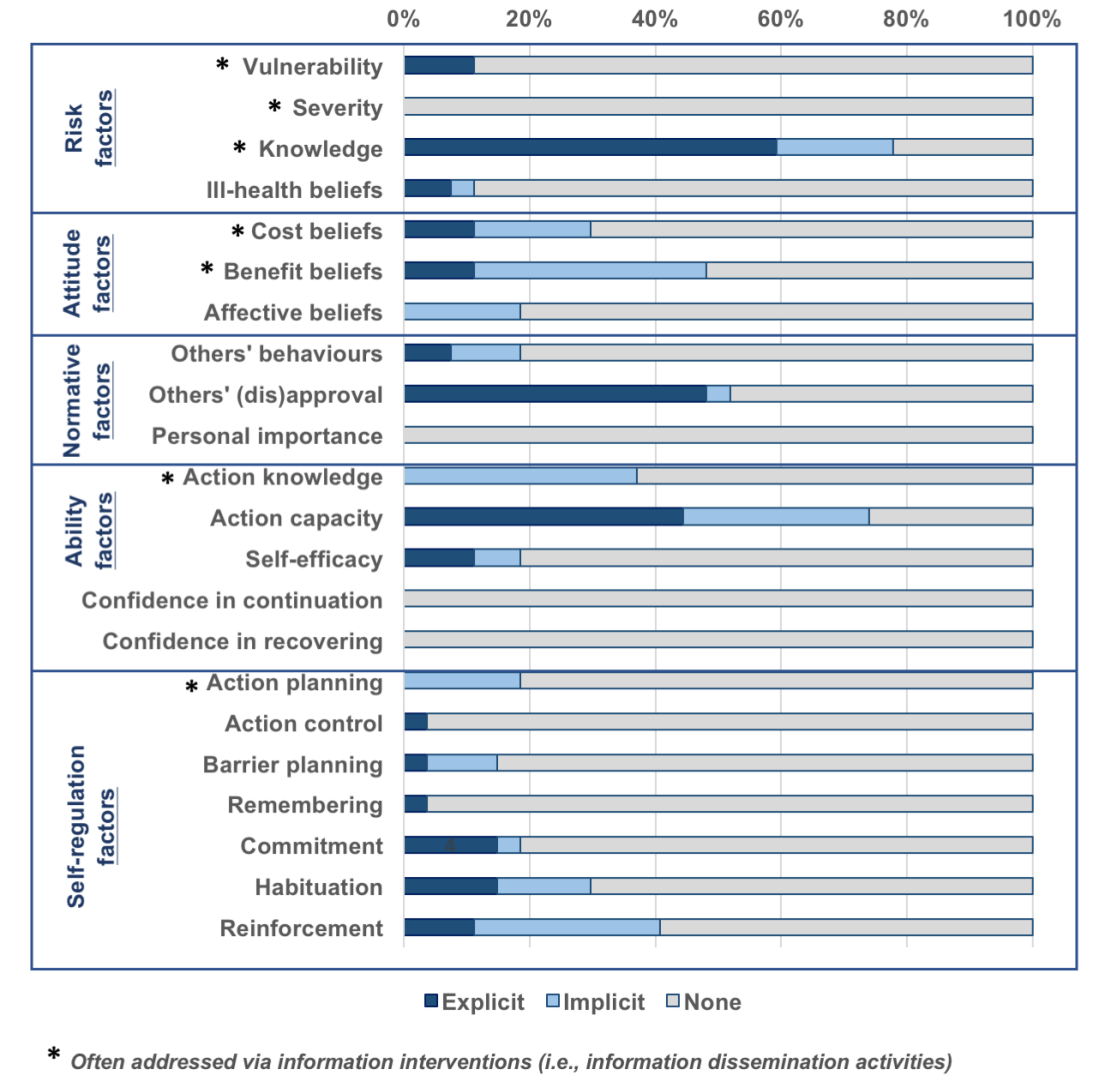
Proportion of endorsed F&E interventions addressing antecedent behavior change factors.

F&E interventions often focus on addressing certain risk and ability factors (Fig 3). Health and hygiene knowledge was the most frequently cited behavior change factor addressed by F&E-related interventions highlighted in the grey literature. Twenty-one (78%) reviewed sources cited these types of information-based components as aspects of their F&E-related interventions (16 sources explicitly, 5 sources implicitly). Action capacity was the second most commonly cited (aggregate) behavioral factor, with 20 (74%) reviewed documents endorsing the incorporation of interventions that address this factor. This is encouraging, as action capacity addresses a more proximal factor of behavior change than knowledge improvement by working to enhance the skills and/or resources necessary to put improved F&E behaviors into action. Perceptions regarding practices typically approved or disapproved of by others, otherwise referred to in the literature as “social norms” (52%, n = 14), and benefit beliefs (48%, n = 13) were other commonly cited proximal factors.

As indicated in Fig 3, none of the included literature endorsed F&E interventions that address two essential ability-related behavioral factors–those related to confidence in continuation and confidence in recovering. Related content and delivery would address one’s perceived ability to re-introduce improved F&E practices into daily life after a disruptive event/stimulus or personal setback.

#### Summary of conceptual and behavioral frameworks indicated in the F&E grey literature

Seven (26%) of the documents included in the review cited behavioral frameworks upon which their interventions and/or assessments were grounded. S4 Table indicates the frameworks presented, and provides a brief summary of each. Two documents each drew on two different frameworks. Consequently, a total of eight frameworks were indicated. While many of the frameworks present different conceptualizations of the behavior change process and antecedents thereof, nearly all of the frameworks acknowledge that individual and community level knowledge and perceptions are important drivers of the change process. Similarly, several of the frameworks stress that the change process should commence at the grass-roots (i.e., community) level, and be driven or led by the community (e.g., PHAST, CLTS, Positive Deviance approaches).

## Discussion

Until recently, major limitations of F&E programming included a lack of behavior-centered F&E content and delivery, and more broadly, insufficient integration of behavior change theory and evidence into the design and implementation of related interventions. Our findings indicate that many F&E-related interventions do not align well with learning from the behavioral sciences [13, 64–67]. This may explain, in part, why such interventions do not yield sustained improvements in F&E behaviors and practices.

Our review demonstrated that a high proportion of documents endorse F&E interventions that focus on knowledge generation via health and hygiene information dissemination and other techniques that address more intermediate and distal behavior change factors. Evidence suggests that information dissemination does not motivate sustained behavior change in the absence of activities that address more proximal change factors, such as improved action capacity (i.e., performance skills), and enhanced attitudes, perceptions, normative beliefs, and self-regulation [64, 68–71]. Our findings reflect a belief from parts of the trachoma community that promotion of eye health via education will serve to achieve behavioral and environmental changes [58]. As indicated in Fig 2, F&E intervention activities endorsed in the literature also overlook factors that are more influential to uptake and sustained adoption of improved practices. The relative deficiency of F&E intervention activities that address more proximal antecedent factors of sustained behavioral change may limit the impact of these interventions.

In addition to several other behavior change frameworks, the RANAS approach suggests that various intervention techniques are necessary for the habitual formation of new behaviors and practices [13, 66, 72]. Wood & Neal [67] suggest that a two-pronged approach of breaking unhealthy habits while simultaneously establishing healthy ones is optimal for facilitating enduring behavior change. Such frameworks include intervention components that move beyond information dissemination to address factors related to behavioral maintenance and resilience.

The majority of identified barriers to improved F&E adoption documented in included literature reflect proximal psychosocial factors. However, F&E-related interventions have largely focused on information dissemination and water and sanitation infrastructure development: activities addressing more distal psychosocial, technological, and contextual factors. While the trachoma community has identified many barriers and facilitators of improved F&E adoption (see Figs 2 and 3), determinants are often either not addressed, or are confronted via inappropriate intervention techniques. A high proportion of documented F&E behavioral determinants reflected barriers, which suggests there is a need to further identify and leverage facilitators of improved behaviors and practices. Enhancements in formative assessments of behavioral determinants might include the performance of motive analyses [73], which aim to identify meaningful facilitators of change.

Intervention content and delivery approaches that support behavioral maintenance and resilience are absent from the F&E programming we reviewed. None of the documents included in this review explicitly or implicitly touch on behavioral maintenance or resilience, key factors facilitating sustained behavior change (Fig 3 and S3 Table) [13, 67]. Maintenance of new behaviors is an important step in habituation. Similarly, resilience is central to sustained adoption of improved F&E practices because it focuses on one’s perceived ability to recover from setbacks and continue the improved practices after disruptions. Recovery from relapse and setbacks is actually part of the behavioral change process, and when appropriately addressed, can serve to facilitate sustained change. Interventions that do not address these dimensions of ability factors often result in only temporary change that is susceptible to relapse [70, 74]. This finding underscores the limited scope of F&E interventions as well as a lack of emphasis on behavior-centered content and delivery, the outputs of which can more readily facilitate sustained change. As such, it should not be surprising to observe behavioral slippage (i.e., regression back to unimproved practices) in areas where initial F&E improvements were observed [75, 76].

Current promising practices from the behavioral sciences and WASH sectors call for sanitation and hygiene programming that:

- is informed by formative research which pinpoints key antecedents and contextually-specific determinants of behavioral change;
- employs a combination of intervention techniques addressing perceived risks, attitudes, normative, and ability factors required to translate improved knowledge and behaviors into practice; and
- includes intervention techniques addressing maintenance-related ability and self-regulation factors that facilitate habitation and sustainability of improved behaviors and practices [13, 63, 65].

Similar approaches can be employed by the trachoma community to design and implement F&E interventions.

Implications of our findings are far-reaching, in that they touch on both the effectiveness and sustainability of F&E interventions. If F&E interventions do not address proximal behavior change factors in ways that bring about and sustain adoption, they may be ineffective. Therefore, it is important for implementers and researchers to assess the composition of intervention content and approaches to delivery. All relevant stakeholders should ensure that proximal behavior change factors are included in their intervention designs, and contextually adapted, as appropriate. Focus should be placed on an intervention mapping and design approach that leverages formative assessments, employs a combination of intervention techniques at different levels of influence, and incorporates intervention components that address behavioral maintenance and resilience.

Our findings corroborate evidence from prior investigation that community-based F&E interventions have largely focused on hardware and resource provision plus information dissemination [77–80]. However, a few works highlight interventions that incorporate approaches or capitalize on non-health motives that address more proximal influencers of improved F&E practices [81, 82]. With regard to the influence of health education interventions, evidence provided by Simms and colleagues [82] suggests that no health education is necessary for the uptake of E-related behavioral outcomes (i.e., coverage, operation and maintenance, and utilization of household latrines), particularly when the intervention affects contextually important benefit beliefs and non-health motives, such as pride and embarrassment.

Well-designed and executed interventions provide evidentiary support that participatory strategies can effectively change facewashing practices [81]. Such interventions reinforce improved behaviors and practices, facilitate communal commitment, and incorporate barrier identification and action capacity activities at the community, group, and individual levels. Other interventions incorporate more proximally influencing social constructs (such as social solidarity) in training guides to harness mutual responsibility and trust to improve utilization of personal and common pool resources (e.g., water) [83]. While some evidence suggests well-designed interventions may be effective when implemented over a short time horizon [81], behavior change and maintenance of improved practices involves a protracted process of confronting underlying behavioral antecedents and determinants. Addressing underlying factors of behavior is not typically accomplished in a sustainable manner over a three to eight month period. This idea is supported within the trachoma community, as some note that intervention activities carried out in the months surrounding mass treatment with azithromycin do not address normative factors (e.g., social disapproval) in such a timeframe [84].

Our work has some limitations. First, the scope of our review was relatively narrow, as we restricted our review to documents posted on key trachoma websites, published in a grey literature database, or submitted to our team by ICTC and WHO-CCT member organizations. While our electronic searches were systematic, they may not have captured all published F&E-related grey literature. We note that although our search dates back to 1965, no documents produced earlier than 1990 were identified. This is perhaps an artifact of website content and document archives. However, the entities engaged represent the majority of stakeholders working on F&E in trachoma elimination programming. Second, in contrast to a systematic review of the peer-reviewed literature, in which reviewers ideally identify and extract data from all eligible papers to reduce risk of biasing the results, the veracity of our review depends on the range of concepts presented, and whether they would be in agreement with those presented in non-reviewed grey literature [34]. As such, we aimed for ‘conceptual saturation’ as opposed to exhaustive discovery, and limited data extraction to intervention approaches endorsed in the literature. However, there is uncertainty regarding the validity of these results beyond the confines of the reviewed documents. Third, the frameworks that we used, particularly RANAS and *Theory of Triadic Influence*, are specific and reductionist in the sense that they focus on psychosocial behavioral factors, and do not fully incorporate contextual and environmental dimensions. A review grounded in other frameworks may yield slightly different results. Finally, we do not have a comprehensive synthesis of empirical evidence that the distal and proximal influencers of behavior that we postulate here are specifically relevant in the context of trachoma.

While acknowledging that some progress has been made in the context of certain funded F&E-related programming, based on the results of this review, we recommend the following:

- Policy-makers and implementers of trachoma elimination programs should critically review the content and delivery of F&E interventions to determine the types of activities and intervention techniques being implemented, and the behavioral factors being targeted.
- Refinements to the design, planning, and implementation of F&E-related interventions should draw on formative assessments aimed at identifying potential mechanisms of change. Intervention mapping or a similar approach should be employed in order to identify a variety of appropriate intervention techniques, with sufficient emphasis on behavioral maintenance.
- Resulting F&E content and delivery should be behavior-centered, leveraging evidence-based motives and aligning with and addressing a variety of known behavioral antecedents and determinants, particularly proximal influencers of change.

## Supporting information

S1 Checklist PRISMA checklist. (PDF)

S1 Appendix Supplemental material. (PDF)

S2 Appendix F&E intervention content & delivery mapping exercise data. (PDF)

S1 Fig Adapted Theory of Triadic Influence. (TIF)

S1 Table Example Boolean search strategy employed during electronic searches of grey literature databases. (PDF)

S2 Table Keyword search terms. (PDF)

S3 Table Antecedent behavior change factors addressed in the grey literature. (PDF)

S4 Table Conceptual and behavioral frameworks indicated in the grey literature. (PDF)
