# Supplementary material for: Interventions to maximize facial cleanliness and achieve environmental improvement for trachoma elimination: A review of the grey literature
Source: PLoS Negl Trop Dis. 2018 Jan 25;12(1):e0006178. doi: 10.1371/journal.pntd.0006178 (PMC5800663; doi:10.1371/journal.pntd.0006178)
Supplement: S1 Appendix — (PDF) [file pntd.0006178.s002.pdf]

S1 Appendix. Supplemental material

Supplemental introduction

Although numerous behavior change intervention techniques exist, some are more successful at eliciting change than others. For example, some techniques operate at a more distal level of influence, and are therefore less likely to facilitate change when implemented in isolation. Others operate at a more proximal level of influence, and are more likely to bring about change. According to the *Theory of Triadic Influence* (S1 Fig), various tiers of influence exist, and account for factors that have direct and indirect effects on behavior and practice [1]. The lowest and most influential tier (i.e., the proximal level of influence) includes factors such as efficacy (e.g., self-efficacy, collective efficacy), attitudes, and social normative beliefs, which directly influence intentions or decisions to act (or not act) in a certain manner. More distal tiers include factors related to knowledge expectancies, values, motivations to comply, and only moderately influence behavior change. Finally, the ultimate level of influence encompasses factors related to biologically and personality-informed traits, as well as cultural, social, and family environments (i.e., enabling environments). These factors indirectly influence behavior.

Supplemental methods details

Additional details related to the search strategy

S1 Table provides an illustrative example of the Boolean search strategy we employed for our search of electronic databases. As various electronic databases have their own respective search nomenclature, this is only an illustrative example, and does not reflect all iterations of the search strategy for all electronic databases that were searched for this systematic review. S2a Table reflects the list of key words we included in our review protocol. These search terms are organised by the key word with which they are most closely related. S2b Table provides a list of search terms that we actually used to search key websites. During an initial round of website searches, we found searching the additional terms in S2a Table did not generate additional documents that were relevant to this review. In other words, searching the terms reflected in S2b Table returned nearly the same volume of relevant results, in a fraction of the time, as searching the more exhaustive list of terms reflected in S2a Table.

Additional details regarding organizations targeted for key website search, literature solicitations

We attempted to target the following International Coalition for Trachoma Control (ICTC) and WHO Coordinating Center for Trachoma (WHO-CCT) member organizations for the search of organizational websites and literature solicitation:

- Adventist Development and Relief Agency (ADRA)
- African Medical and Research Foundation (Amref)
- Blantyre Institute for Community Ophthalmology (BICO)
- CARE
- cbm
- Johns Hopkins Center for Communication Programs
- Emory University
- Eyes of the World
- Heart to Heart Foundation
- Helen Keller International (HKI)
- IMA World Health
- International Agency for the Prevention of Blindness (IAPB)
- International Trachoma Initiative (ITI)

- International Coalition for Trachoma Control (ICTC)
- Kilimanjaro Centre for Community Ophthalmology (KCCO)
- Kongwa Trachoma Project
- Light for the World
- Lions Clubs International Foundation
- Live & Learn
- London School of Hygiene and Tropical Medicine
- Magrabi Foundation
- Organisation pour la prevention de la cecite (OPC)
- Operation Eyesight Universal
- Orbis International
- RTI International
- Sightsavers
- The Carter Center
- The Fred Hollows Foundation
- The University of Melbourne
- Wake Forest School of Medicine
- WaterAid
- Wilmer Eye Institute at Johns Hopkins
- World Vision International
- Yonsei University Severance Hospital
- University of California San Francisco – Francis I. Proctor Foundation

Additional details related to eligible interventions

While evidence regarding the impact of the following interventions on trachoma outcomes is uncertain, we included them in our list of eligible interventions given they are often endorsed as interventions to address environmental improvement:

- Animal husbandry practices (e.g., increasing distance between animal pens and the homestead, safe animal faeces disposal); and
- Waste management (e.g., improved solid and liquid waste disposal).

Additional details related to mapping of endorsed F&E-related intervention content

In addition to information related to document identification and publication, we mapped the following five categories of characteristics and their accompanying attributes (i.e., data items) from those documents included in the review of the grey literature:

1. Type of document
2. Purpose of document
3. Intervention content (i.e., components, activities)
    a. Intervention design
    b. Intervention activities
    c. Determination of whether intervention activities address known barriers
4. Implementation delivery (e.g., approaches, strategies)
    a. Type and level of implementation
    b. Proposed/endorsed conceptual/behaviour change frameworks, behaviour change techniques
5. Monitoring and evaluation methods
    a. Metrics
        i. Types of metrics
        ii. Proposed F and E indicators

Supplemental results details

The 16 F&E-related intervention activity themes identified through our thematic analysis, and reflected in the rows of Fig 2 are presented below. We categorized the intervention themes against the RANAS intervention techniques in the list below. However, as a result of organizing the intervention categories by RANAS intervention technique, the grouping of intervention themes below does not align exactly with the clustering of rows in Fig 2. We ordered the rows in Fig 2 strictly according to level of influence, without regard to the RANAS intervention technique classification.

<u>Enabling environment-related intervention activities</u>

- Inclusion of F&E in national curricula, infrastructure, policy
- Planning, executing advocacy strategies that address F&E-specific barriers
- Establishing partnerships, meeting with stakeholders involved in F&E/WASH programming

<u>Information intervention activities</u>

- Collecting information to develop F&E/WASH situation analyses, identify barriers of F&E-related behaviour change, track progress (e.g., community assessments – mapping of community resources, facilities [e.g., water sources, community and household latrines, face-/handwashing stations], and disease)
- Changing knowledge of trachoma transmission and prevention, including F&E-related preventive behaviours, beliefs
- Disseminating or collecting data to change perceived risk of trachoma
- Collecting data to improve the understanding of social and cultural determinants of trachoma, F&E-related behaviours and practices
- Increasing community engagement to identify barriers to trachoma, improved F&E behaviours and practices

<u>Infrastructural & ability intervention activities</u>

- Improving access to, availability, and proper utilisation of resources necessary to carry out improved F&E behaviours (e.g., CLTSH, latrine promotion, water point and/or latrine installation)
- Improving required skills and opportunities for carrying out improved F&E practices
- Changing perceptions regarding self-efficacy of improved F behaviours and practices (individual-and household-level), collective efficacy regarding improved E behaviours (household and community-level)

<u>Persuasive intervention activities</u>

- Introducing F&E, behaviour change messages and/or promotional activities via various community-level entry points
- Changing attitudes toward improved F&E-related behaviours
- Changing perceptions regarding the amount of water needed for washing one's face and hands

<u>Normative intervention activities</u>

- Changing "norms" to be consistent with improved F&E practices (e.g., changing beliefs regarding dirty faces such that people perceive that others think they ought to wash their children's faces and their own faces – i.e., normative expectations; people perceive that others actually do wash their children's faces and their own faces - i.e., empirical expectations).

<u>Planning & relapse prevention</u>

- Reinforcing improved F and E practices

References

1. Flay, B.R. and J. Petraitis, *The Theory of Triadic Influence: A new theory of health behavior with implications for preventive interventions.* Advances in Medical Sociology, 1994. **4**: p. 19-44.
