# Supplementary material for: Interventions to maximize facial cleanliness and achieve environmental improvement for trachoma elimination: A review of the grey literature
Source: PLoS Negl Trop Dis. 2018 Jan 25;12(1):e0006178. doi: 10.1371/journal.pntd.0006178 (PMC5800663; doi:10.1371/journal.pntd.0006178)
Supplement: S2 Appendix — (PDF) [file pntd.0006178.s003.pdf]

S2 Appendix. F&E intervention content & delivery mapping exercise data

F&E-specific grey literature

<u>Health promotion partnerships for trachoma elimination</u>

- **Type of document:** Implementer report/exchange intended to provide information on a spectrum of health promotion strategies for engagement, advocacy, and the elimination of trachoma in the Northern Territories of Australia
- **Purpose of document:** None stated, but inferred purpose is to highlight the role of health promotion in increasing the focus of elimination of trachoma on sustainable improvements facilitated through F and E components of the SAFE strategy
- **Intervention components/activities:** Working on enabling environments by: 1) ensuring key messages are consistent and hygiene practices are mandated by including them in national curricula, health infrastructure, and policy responses; 2) increasing understanding of social & cultural determinants and generating practical, evidence-based, community-led policy responses; and 3) translating knowledge & practice into upstream socio-environmental approaches. Working at the community level to: 1) increase community engagement to identify barriers to trachoma elimination; 2) encourage everyone to help young children wash their faces whenever nose/eye secretions are present; and 3) ensure morning facewashing, nose blowing, handwashing, and tooth brushing to enable children to be 'school ready', promote health, and facilitate good participation in class.
  - **Design:** Cross-sectoral collaboration, community engagement & community-led implementation
  - **Do these activities address known barriers:** Yes, resource suggests engaging community to identify social & cultural determinants of trachoma as well as barriers to trachoma elimination
- **Implementation approach/strategies:** Employing medical, behavioural, and socio-economic approaches via multi-faceted health education strategies and resources to incorporate 2 key F&E-related messages (i.e., "clean faces, strong eyes" & "safe & functional bathrooms") into programming at numerous entry points within the community setting (e.g., cultural events, schools, early child & family well-being settings, clinics)
  - **Type and level of implementation:** Designing, planning, implementation, monitoring at the national, sub-national, community, school (including early child & family well-being settings), clinic, and individual levels
  - **Proposed behaviour change frameworks and/or techniques:** No particular frameworks mentioned
- **Monitoring & evaluation methods:** Document does not discuss monitoring/evaluation methods
  - **Metrics:** None mentioned
    - **Types of proposed metrics:** None mentioned
    - **Proposed F&E Indicators:** None mentioned
- **Tools included:** None

<u>ICTC – All you need for F&E</u>

- **Type of document:** Technical guidance for design, implementation, monitoring/evaluation of F&E interventions
- **Purpose of document:** Planning resource for all partners supporting national trachoma programmes that provides planning approaches and tools to coordinate WASH as part of integrated SAFE programmes for achieving elimination of trachoma by 2020
- **Intervention components/activities:**
    - **Design:** Resource provides guidance on how to assess the context in which the F&E interventions will be incorporated and use data from national, sub-national, local, and community levels to design F&E interventions that are meaningful in the local context
    - **Do these activities address known barriers:** Resource provides guidance on how to assess barriers to uptake of improved practices, and how to design interventions to address these issues (no specific barriers identified or suggested)
- **Implementation approach/strategies:** Creating and fostering cross-sectoral partnerships/collaboration, developing and implementing F&E interventions that facilitate related and sustainable behavioural change, planning and monitoring of F&E activities and outcomes/sustainability of outcomes
    - **Type and level of implementation:** Designing, Planning, Implementing – National to community levels
    - **Proposed behaviour change frameworks and/or techniques:** No specific behaviour change frameworks or techniques promoted, but resource does encourage the generation of data on the following to inform F&E-related interventions that facilitate behavioural change and habit formation: knowledge/perceptions, skills, social norms, access to resources that facilitate improved practices
- **Monitoring & evaluation methods:** Resource suggests programme M&E frameworks align with national and sub-national frameworks, but include additional indicators, as needed
    - **Metrics:**
        - **Types of proposed metrics:** individual, household, community, school, and process indicators suggested (no specific suggestions with regard to measurement methods – e.g., respondent-reported, directly observed)
        - **Proposed F&E Indicators:**
- **Tools included:**
    - Planning tool/checklist
    - Behaviour change worksheet
    - Principles for F&E
    - Cross-sectoral meeting for full F&E implementation: Annotated agenda
    - Targeted advocacy messages
    - Situation analysis terms of reference template
    - F&E planning and design workshop agenda
    - F&E planning worksheet
    - F&E report template

<u>Trachoma action planning: A planning guide for the national elimination of blinding trachoma</u>

- **Type of document:** Technical guidance for developing an effective and efficient trachoma action plan that attends to the entire SAFE strategy

- **Purpose of document:** To provide guidance on setting aggressive but realistic targets and determining steps required to develop a tangible trachoma action plan
- **Intervention components/activities:** Determine specific F&E activities needed to enable the elimination of active trachoma, including: compiling data on water and sanitation access and utilisation (coverage used in the document), convening planning meetings with those involved in F&E (e.g., WASH officials, NGO partners), compiling information on trachoma and WASH activities at the district level, identifying F&E partners, conducting a full F&E situation analysis, cataloguing existing hygiene/BCC programmes/events
    - **Design:** Resource provides guidance on how to design a trachoma action plan that includes F&E-related interventions/activities
    - **Do these activities address known barriers:** The resource includes a tool on F&E and coordination questions that encourages participants to question and discuss whether water availability is a barrier to facewashing, and if so, to discuss what creative strategies exist as an alternative
- **Implementation approach/strategies:** Action planning, including the identification of F&E partners, completion of a full F&E situation analysis, cataloguing of existing hygiene/BCC programmes/events
    - **Type and level of implementation:** Planning – National to district levels
    - **Proposed behaviour change frameworks and/or techniques:** None suggested, though the resource advises programme implementers on the cataloguing of existing hygiene/BCC programmes and events
- **Monitoring & evaluation methods:** None suggested, though the resource does indicate the following data on F&E interventions should be collected: reports on specific F&E activities, "information on clean face" prevalence, and the indicators included in the F&E situation analysis tool
    - **Metrics:** Resource proposes collation of data on district-level water and sanitation coverage, hygiene activities undertaken (e.g., handwashing) and environmental improvement activities (e.g., water access, latrines) by partner
        - **Types of proposed metrics:** District-level, participant-reported process and outcome (i.e., coverage, access) indicators
        - **Proposed F&E Indicators:** Resource mentions district-level water and sanitation coverage, and non-specific hygiene and BCC process indicators
- **Tools included:**
    - F&E worksheet
    - Full F&E situation analysis guide
    - F&E and coordination questions

<u>**Education and trachoma**</u>

Type of document: Implementer report (poster)

Purpose of document: To explain the important link between trachoma elimination and education, outline some key definitions, and provide some background information about the role of schools and early childhood settings as central to improving hygiene practices

- **Intervention components/activities:** Community health programmes involving many groups, such as early childhood services, family services and centres, and schools to improve hygiene (i.e., children must blow their nose, wash their faces and hands, and brush their teeth to be "school

ready" therefore removing eye/nose secretions); school policy reinforcing new skills and good hygiene – e.g., facewashing is included as a competencies in the *Assessment of Student Competencies*; provision of hygiene products within health promotion activities; establishment of a portal to "up-skill" teachers including the sharing of digital resources
    - **Design:** School and community-based hygiene promotion
    - **Do these activities address known barriers:** Yes, barriers related to social norms, skills, resources, and hygiene facilities
- **Implementation approach/strategies:** Integrating facewashing and improved hygiene promotion in school curricula; establishing clean faces as the new social norm; ensuring there are safe and functional washing facilities that are properly maintained with access to water, soap, paper towels, tissues, mirrors, and bins; ensuring facewashing is part of the curriculum and is implemented in remote schools with encouragement and support for every day hygiene practices; utilising Trachoma Story Kit resources for schools to raise awareness of teachers and have activities for children about the importance of good hygiene for health and well-being
    - **Type and level of implementation:** Planning and implementation – local, school, and community levels
- **Proposed behaviour change frameworks and/or techniques:** Behaviour change messaging/campaigns: Key messages for service/centre/school settings: "Clean faces, strong eyes" and community settings: "Clean faces, safe bathrooms"; modelling behaviours and side-by-side engagement and discussion in culturally safe and supportive family service mechanisms (in pre-school centres e.g., family wellbeing centres, play groups, child care, and other early childhood services); provision and maintenance of facilities (e.g., accessible, safe, and clean washrooms) and materials children need to ensure their faces and hands are clean (e.g., provision of soap, paper towels, tissues, bins, and installation of large safety glass mirrors so children can see if their faces are clean, training of school attendance officers to ensure children are "school ready with clean faces"; forging partnerships between schools and pre-schools in remote communities to develop collaborative health promotion initiatives with early childhood cenres, visiting health and education services, sports groups, and arts-based NGOs.
- **Monitoring & evaluation methods:** Document does not discuss monitoring/evaluation methods
    - **Metrics:** None mentioned
        - **Types of proposed metrics:** None mentioned
        - **Proposed F&E Indicators:** None mentioned
- **Tools included:** None

<u>Formative research assessment and guidelines: Facial cleanliness and environmental sanitation</u>

- **Type of document:** Technical guidance for the implementation of F&E-related formative research
- **Purpose of document:** To collect evidence about trachoma and the factors and practices that promote transmission of infection that have potential to impact the effectiveness and success of prevention programmes; to increase understanding of the local context and inform decision-making regarding programme approaches and strategies for promoting behavioural change
- **Intervention components/activities:** Community visits and walk-throughs, informal interviews, focused data collection that aligns with WASH-IBM
    - **Design:** Mixed-methods formative research
    - **Do these activities address known barriers:** Resource is designed to guide programme implementers in identifying context-specific barriers (and drivers) of behaviour change

- **Implementation approach/strategies:** Community visits, interviews, focused data collection
    - **Type and level of implementation:** Data generation and collation – National to individual levels – collation of national and district level data, generation of qualitative and quantitative data at community, household, and individual levels
    - **Proposed behaviour change frameworks and/or techniques:** Resource encourages the use of an assessment approach based on a conceptual framework that acknowledges multi-level, inter-related factors influencing WASH-related decisions and behaviours amongst community groups and individuals. Adaptations of the IBM-WASH Framework and the Communication for Water Treatment and Hygiene Framework are highlighted. Specific techniques suggested include modification of F&E-related perceptions regarding disease transmission, risk of disease, attitudes toward improved behaviours, self-efficacy, and subjective norms
- **Monitoring & evaluation methods:** Situational assessment
    - **Metrics:** Questions aimed at facilitating data elicitation and collation aligned with an adaptation of the WASH-IBM and Communication for Water Treatment and Hygiene frameworks that are tailored toward trachoma prevention
        - **Types of proposed metrics:** Input, process, outcome indicators; respondent-reported, directly observed, and objective indicators
        - **Proposed F&E Indicators:**
- **Tools included:** F&E focused data collection tool (adaptation of IBM-WASH tailored for generation/collation of data to design F&E interventions for trachoma prevention)

<u>Protocol and methods for trachoma situational analysis: Using a systematic process for understanding F&E for trachoma programs</u>

- **Type of document:** Technical guidance for performing an F&E-centric trachoma situational analysis
- **Purpose of document:** To review the national health goals, policies, strategies, and plans; assess the strengths and weaknesses of the institutional support systems; assess the health service design and implementation strategies at the national and district level; and assess the flow of information and implementation materials to and from the national and district level
- **Intervention components/activities:** National level assessment of the contribution of national health and WASH policies and other relevant health management systems or health services that create enabling environments (at national and district operational levels) that allow for effective implementation of trachoma control, hygiene, and sanitation interventions.
    - **Design:** Situational analysis 'checklist' of topics that may be used to further guide F and E programming and activities within the context of national trachoma programmes. Resource encourages collection of information related to the following topics:
        - Demographic, climate, disease prevalence
        - Trachoma and WASH coordination
        - Trachoma and WASH tools
        - Advocacy
        - Media channels
    - **Do these activities address known barriers:** Resource does not present information on barriers, but does suggest tapping into local knowledge from the government and

understanding communities needs and preferences for the creation of suitable F&E interventions

- **Implementation approach/strategies:** Data/information collation, networking/collaboration
  - **Type and level of implementation:** Design, planning – National and district levels
  - **Proposed behaviour change techniques:** None mentioned
- **Monitoring & evaluation methods:** No specific methods suggested, though the situational analysis does draw on data most likely collected via other (national or district-level) M&E frameworks
  - **Metrics:** Resource proposes no specific F&E metrics
    - **Types of proposed metrics:** Resource does advise collating national and district-level data; respondent-reported and directly observed indicators identified
    - **Proposed F&E Indicators:** district-level data on access to improved water, access to improved sanitation facility, proportion of population practicing open defecation, current rates of TF, TT, dirty face, MDA coverage, population densities around water sources, number of sanitation facilities available, functionality of water and sanitation facilities and management committees, drought risk, rate of acute respiratory disease, population mobility (e.g., nomadic, pastoralist)
- **Tools included:** None, though this resource is complemented by the document entitled *Formative research assessment and guidelines: Facial cleanliness and environmental sanitation.*

<u>Understanding individual and contextual factors for development of a behavior change communication campaign for trachoma prevention in Busoga and Karamoja regions, Uganda</u>

- **Type of document:** Implementer report – presentation of findings from formative research intended to guide the development of an F&E behaviour change communication campaign
- **Purpose of document:** To present findings from formative research conducted to identify and understand the contextual and behavioural factors that influence the transmission and prevention of trachoma in study sites; to characterise factors influencing community members' ability to implement F&E components of the SAFE strategy; and provide information to support the development of an F&E BCC campaign
- **Intervention components/activities:** Formative research intended to identify individual and contextual factors for developing a behaviour change campaign for trachoma prevention
  - **Design:** Mixed methods – qualitative data collection via in-depth interviews and household observations, quantitative data via household survey and observations
  - **Do these activities address known barriers:** Resource presents findings from a formative research activity designed to identify relevant communication strategies intended to address barriers to and facilitators of behaviour change
- **Implementation approach/strategies:** Mixed methods formative research intended to provide information to inform the development of an F&E BCC campaign
  - **Type and level of implementation:** Data generation, design, planning at the community level
  - **Proposed behaviour change frameworks and/or techniques:** The research described herein is grounded in the Risk Perception Attitude Framework (RPA), adapted from the Extended Parallel Process Model (EPPM). The Communication for Water Treatment and Hygiene Framework is also mentioned. This framework is intended to be a predictive model to guide the design and evaluation of interventions to promote good WASH

behaviours. Resource describes a theory-based approach to behaviour change communication programme design to promote the use and scale-up of best practices, and improve outcomes and country-level advocacy for trachoma prevention

- **Monitoring & evaluation methods:**
    - **Metrics:** F&E-related attitudes (knowledge, beliefs, values), perceived risk, perceived control (self-efficacy), norms, and environmental factors (e.g., availability and access to clean potable water, coverage of community-based facilities for waste disposal, fly density, household latrine coverage, proximity of animals, open defecation)
        - **Types of proposed metrics:** Antecedents of behaviour change; respondent-reported, directly observed and objective measures
        - **Proposed F&E Indicators:** No specific F&E indicators recommended
- **Tools included:** None

<u>**ICTC Principles for F&E**</u>

- **Type of document:** Technical resource to inform programming and partnerships for F&E intervention implementation
- **Purpose of document:** To guide decisions on programme partnerships, planning, and design for ICTC members; and, more generally, to serve as a resource for policy makers within national governments of endemic countries, donors supporting trachoma elimination/NTD initiatives, and affected communities
- **Intervention components/activities:** Resource outlines numerous activities related to 10 key F&E principles
    - **Design: Resource presents 10 F&E principles:** 1. Country ownership of national programmes; 2. Sustainability; 3. Partnership and participation; 4. Equity, inclusion, and non-discrimination; 5. Integration; 6. Use of evidence; 7. Joint advocacy; 8. Context-driven social and behavioural change; 9. Tracking progress and measuring success; 10. Viable financing
    - **Do these activities address known barriers:** Resource suggests local contexts (e.g., contextually specific barriers) should be considered when designing and implementing F&E interventions
- **Implementation approach/strategies:** Resource suggests programmes and interventions should be community-centred via active engagement of affected communities in planning, decision-making, and relevant training, with explicit efforts to involve marginalised people
    - **Type and level of implementation:** Design, planning, implementation (including M&E) – National to community levels, with focus on action at national to district levels
    - **Proposed behaviour change frameworks and/or techniques:** No specific BC techniques indicated or suggested, but the resource does indicate that social and behaviour change is a key element of sustainability. One specific principle – context-driven social and behaviour change – highlights the need to address social norms and behaviours in sustained and culturally acceptable ways based on a robust understanding of the specific contexts in which F&E interventions are delivered and taking into account social, cultural, economic, geographic, and resource considerations
- **Monitoring & evaluation methods**: No specific methods suggested, though the resource does suggest that operational research and evaluations are needed to contribute to building and

strengthening evidence bases on effective practices, and collection of data at national and district levels is needed to track progress against global indicators and national milestones
    - **Metrics:** Resource proposes no specific F&E metrics
        - **Types of proposed metrics:** National and district-level input, process, outcome, and impact data
        - **Proposed F&E Indicators:** None proposed
- **Tools included:** None included

<u>Research to inform the development of behaviour change interventions for "F" and "E" of the SAFE strategy in Turkana and Marsabit, Kenya</u>

Type of document: Implementer report – presentation of findings from formative research intended to guide the development of behaviour change interventions for F&E

- **Purpose of document:** To present findings from formative research intended to establish the factors influencing community behaviour and practices that perpetuate the spread of trachoma; to understand the attitudes, practices, and behaviours of communities affected by trachoma in Turkana and Marsabit, Kenya with respect to water use, hygiene and sanitation, and health seeking behaviour
- **Intervention components/activities:** Mixed methods formative research
    - **Design:** Mixed methods – qualitative data obtained via in-depth interviews and focus group discussions; quantitative data obtained via structured observations (household compound, community water collection points), facial cleanliness assessments, environmental spot checks, household demographic and knowledge, attitudes, and practices surveys
    - **Do these activities address known barriers:** Resource identifies the following barriers, and notes that the resulting F&E interventions should consider the physical and social environment and tap into drivers of [improved] behaviour[s]: physical/infrastructural barriers (e.g., lack of water, limited means of transport in most communities, difficulties in building latrines, perceptions that latrines are not a valuable use of time/resources); cultural barriers (e.g., lack of distinction between hygiene and health, presence of nasal discharge as social norm, keeping animals in close proximity to compounds for security reasons)
- **Implementation approach/strategies:** Mixed methods formative research intended to guide the development of behaviour change interventions for F&E
    - **Type and level of implementation:** Data generation, design, planning at the community level
    - **Proposed behaviour change frameworks and/or techniques:** "Evo-Eco" approach – a theoretical framework of the factors influencing behaviour
- **Monitoring & evaluation methods:**
    - **Metrics:** Respondent-reported indicators (e.g., latrine use, location of open defecation,) direct observation and objective indicators (e.g., proportion of defecation events accompanied by handwashing, number of households with water available in the early morning, approximate amount of water available very early in the morning, amount of water collected, person collecting the water, time taken to reach the water source, time spent at the water source, amount of water collected, practices at water points, personal washing practices, handwashing at key times, water use by household activity, facial

wiping, facial cleanliness of young children, personal washing practices including facewashing)
        - **Types of proposed metrics:** Antecedents of behaviour change; respondent-reported, directly observed and objective measures
        - **Proposed F&E Indicators:** No specific F&E indicators proposed
- **Tools included:** None

<u>WASHing away blinding trachoma</u>

Type of document: Policy/advocacy piece for the comprehensive implementation of the SAFE strategy

Purpose of document: To advocate for the integration of F and E components of the SAFE strategy from the outset of trachoma control programmes to ensure that the underlying causes, as well as the symptoms, of the disease are addressed to break transmission cycles

- **Intervention components/activities:** Multi-sectoral collaboration between education, WASH, and health stakeholders (e.g., ensuring, through clear planning, role allocation and monitoring, that the full SAFE strategy is implemented); integration of community-led F&E interventions into trachoma control programmes from the outset; incorporation of disease control objectives into WASH programmes; explicitly targeting programmes toward the poorest and most marginalised communities and ensuring inclusive design and service delivery for people with disabilities
    - **Design:** Cross-sectoral collaboration and integrated, community-led implementation
    - **Do these activities address known barriers:** No specific barriers mentioned, though resource does indicate the need to address environmental, attitudinal, and institutional barriers (specifically those that marginalised populations face)
- **Implementation approach/strategies:** Holistic approach to development that tackles both the symptoms and underlying causes of poverty and ill-health
    - **Type and level of implementation:** Design, implementation, advocacy – global to community levels
    - **Proposed behaviour change frameworks and/or techniques:** Resource does not promote any specific framework
- **Monitoring & evaluation methods:** Resource does not discuss monitoring/evaluation methods
    - **Metrics:** None mentioned
        - **Types of proposed metrics:** None mentioned
        - **Proposed F&E Indicators:** None mentioned
- **Tools included:** None

<u>Clean faces, strong eyes</u>

Type of document: Implementer report/exchange intended to provide information regarding experiences with improving F-related knowledge, attitudes, and practices via health promotion & social marketing

- **Purpose of document:** None stated, but inferred purpose is to highlight the role of health promotion resources and social marketing strategies in improving knowledge, attitudes, and practices regarding improved F-related behaviours
- **Intervention components/activities:** Development and employment of health promotion resources and social marketing strategies that: 1) motivate uptake through the use of social marketing strategies that leverage respected role models; 2) disseminate health promotion

practices by being noticeable and present at key events/times; and 3) reinforce improved F-related practices via repeated and memorable dissemination events.

    - **Design:** Cross-sectoral collaboration, community engagement & community-led implementation
    - **Do these activities address known barriers:** Health promotion resources & social marketing strategies seek to address barriers regarding knowledge amongst clinic, school, and community staff of their awareness that they work in a trachoma-endemic area, and attitudes and norms regarding dirty faces
- **Implementation approach/strategies:** Developing and employing culturally appropriate health promotion resources and social marketing strategies via a consultative process to ensure: 1) the indigenous culture is respected; 2) messages are clear & holistic; and 3) local people, environment, and popular culture are involved.
    - **Type and level of implementation:** Design, planning, implementation at national, sub-national, and community levels
    - **Proposed behaviour change frameworks and/or techniques:** No specific framework mentioned, though the resource indicates the health promotion resources & social marketing strategies are intended to improve knowledge regarding trachoma transmission & prevention, change attitudes and norms regarding dirty faces, and leverage clinic, school, and community staff's comfort regarding talking about hygiene
- **Monitoring & evaluation methods:** Document does not discuss monitoring/evaluation methods
    - **Metrics:** None mentioned
        - **Types of proposed metrics:** None mentioned
        - **Proposed F&E Indicators:** None mentioned
- **Tools included:** None

<u>How communities can control for trachoma without a big budget</u>

Type of document: Policy/advocacy piece; implementation guidance drawing attention to tangible, low-/no-cost options for community engagement in trachoma control

- **Purpose of document:** None stated, but inferred purpose is to inform programme implementers and policy-makers about low-/no-cost ways in which communities and community partners can implement interventions to prevent and control trachoma
- **Intervention components/activities:** Policy/advocacy, intervention design/implementation, including promotion and uptake of individual, household, and community behaviours that prioritise and act to ensure faces are clean, household members (all community members) dispose of their faeces in a safe way, and households and communities are free of material that attracts flies (i.e., waste management systems are in place). Illustrative activities include: promoting facewashing amongst families – especially children; teaching families that sharing towels or cloths can put loved ones at risk of infection if someone has trachoma; ensuring trachoma prevention and hygiene education are taught in primary schools; every household in the community maintains access to a latrine; encouraging community-wide sanitation, including waste disposal (i.e., infant and child faeces disposal, solid and liquid waste, animal waste); encouraging households to build, use, and maintain a household latrine (and share with households that do not have a latrine until they can build their own); conducting community household sanitation surveys, and sharing the data; congratulating families who sweep their compounds every day and keep them clean, and celebrate them as models; making connections

with local communities partners (e.g., local NGOs) that work in water, sanitation, and hygiene to help them target WASH improvements in communities where trachoma is highly prevalent

  - **Design:** Community-based interventions
  - **Do these activities address known barriers:**

- **Implementation approach/strategies:** Intervention design/implementation, including promotion and uptake of individual, household, and community behaviours that prioritise and act to ensure faces are clean, household members (all community members) dispose of their faeces in a safe way, and households and communities are free of material that attracts flies (i.e., waste management systems are in place). Resource suggests that facial cleanliness, hygiene promotion, and access to water and sanitation should be thought of as cornerstones of trachoma prevention – on which antibiotics can be added.
  - **Type and level of implementation:** Design, planning, and implementation at community, household, individual levels
  - **Proposed behaviour change frameworks and/or techniques:** None mentioned
- **Monitoring & evaluation methods:** Resource proposes the administration of community household sanitation surveys and sharing of data with local health clinics, government offices, or NGOs
  - **Metrics:** Community, household, and individual level metrics
    - **Types of proposed metrics:** No specific metrics proposed
    - **Proposed F&E Indicators:** No specific F&E indicators proposed
- **Tools included:** None
- **Notes:** Resource provides a nice breakdown of F and E as well as a good overview of individual/household and community actions that can be taken to prevent and control trachoma.

<u>The end in sight: 2020 INSight</u>

Type of document: Policy/advocacy piece; technical guidelines – global strategic planning and guidance for national level planning for implementation of the SAFE strategy

- **Purpose of document:** A global strategic plan and guide for simultaneous planning at the national level to help mobilise partners and resources to accelerate efforts along a common path to reach the goal of global elimination of blinding trachoma by 2020. Authors indicate this is an advocacy document intended to increase awareness, political will, and funding that seeks to outline the following: a review of the current state of trachoma elimination; an overview of the aspiration, analysis of the interventions needed and examples of paths to success; milestones for progress to 2020 based on overarching principles and a detailed approach to an individual district; and the total cost to eliminate blinding trachoma and the impact of the investment.
- **Intervention components/activities:** Well-planned and balanced implementation of the SAFE strategy, country commitment, collaborative partnerships, engagement of a broader funding, consistent advocacy. More specifically for F&E, the resource mentions the implementation of health education programmes on face washing and the installation of safe water points and household latrines
  - **Design:** Cross-sectoral collaboration with ministries of health, education, water and sanitation, iNGOs, academic institutions, donors, and community partnerships
  - **Do these activities address known barriers:** Resource suggests scale-up of interventions is critical but requires addressing important barriers. Resource also indicates it is important to obtain more detailed information before an appropriate set of interventions

can be designed such that the interventions address contextually-specific barriers such as the presence of nomadic populations or people living in border regions. However, it is acknowledged herein that behaviour change remains a difficult public health intervention to achieve, maintain, and measure.

- **Implementation approach/strategies:** Resource presents 5 guiding principles that provide a framework for the path to elimination: 1. Urgency, 2. Accountable ownership, 3. Integration, 4. Efficient and coordinated partnership, 5. Tailored interventions. Resource indicates that each endemic district should implement a comprehensive approach to ensure community workers, trained teachers, and public leaders are engaged, and the community at large is informed of the benefits of facial cleanliness. The resource encourages the integration of the F component in national handwashing campaigns.
    - **Type and level of implementation:** Planning, implementation – global, national and sub-national (i.e., district) levels
    - **Proposed behaviour change frameworks and/or techniques:** Though behaviour change is mentioned several times throughout the document, no specific frameworks or techniques are mentioned. Resource does mention that it is important to avoid creating new programmes to achieve the F component, but to instead build on existing efforts focused on broader behaviour change.
- **Monitoring & evaluation methods:** No specific M&E methods mentioned, though the document does indicate that a detailed epidemiological survey in all suspected geographies is required
    - **Metrics:** Document does not include a discussion regarding specific types of metrics to be collected
        - **Types of proposed metrics:** No specific metrics proposed
        - **Proposed F&E Indicators:** No specific F&E indicators suggested, though an illustrative example includes water coverage and latrine coverage indicators as indicative of programme success
- **Tools included:** None included
- **Notes:** Resource highlights that behavioural changes that result from F and E interventions can form an effective barrier against resurgence, and as such, it is important to continue to support facial cleanliness, latrine utlisation, and the improvement of water and sanitation infrastructure. Authors indicate inclusion of facial washing in educational programmes and other WASH campaigns and coordination thereof is the key to sustainable elimination. The resource also highlights the fact that F and E interventions will help prevent the transmission of other diseases.

<u>**Trachoma resource book**</u>

Type of document: Technical guide to resource materials for practical support for the implementation of trachoma control activities in Australia

- **Purpose of document:** To pull together some of the current key information about trachoma and its control to assist people involved in trachoma control activities
- **Intervention components/activities:** Health promotion to educate community about trachoma, the importance of personal hygiene, and practical advice on facial cleanliness. Promotion of facial cleanliness through education, targeting both expectant mothers and young children, innovative methods to translate health education and promotion activities into sustainable changes in behaviour, involvement of everyone who interacts with children to promote clean faces (e.g., football coaches, art teachers, swimming pool attendants), promotion of clean faces as a new

community injunctive norm by family and community service workers, positive and non-judgemental reinforcement of messages. Resource suggests that in Australia, environmental improvements should focus on addressing barriers to children washing their faces and achieving facial cleanliness, such as access to water and appropriate use of water for hygiene purposes. Examples include ensuring proper maintenance and repair of housing, especially washing facilities and bathrooms; checking household and community washing facilities to ensure they are functional and safe for children to use; installing mirrors so children can actually see whether their faces are clean.
    - **Design:** Local (i.e., government-run, regional public health units) and community-based intervention implementation
    - **Do these activities address known barriers:** Barriers, as identified by the authors of the document include: lack of access to or inappropriate allocation of clean water for hygiene purposes (e.g., in Australian contexts, poorly maintained or constructed washing facilities or bathrooms, including broken plumbing, missing taps, slippery floors/basins), lack of awareness of the benefits of keeping children's faces clean, time constraints, and lack of motivation.
- **Implementation approach/strategies:** Resource recommends a programme development, implementation, and evaluation approach that involves engagement and partnership with the local community at all levels and includes genuine collaboration and consultation with Aboriginal Community Controlled Organisations, community representatives, and other key stakeholders and experts; increasing collaboration at national, regional, and community levels to ensure comprehensive implementation of the SAFE strategy, and establishing and maintaining a health workforce with appropriate knowledge, skills, and experience in trachoma control through regular training and support
    - **Type and level of implementation:** Design, implementation, monitoring – community and local levels
    - **Proposed behaviour change frameworks and/or techniques:** No specific beahviour change framework noted, but the following techniques were encouraged throughout the resource: changing perceptions regarding the amount of water needed to keep children's faces and hands clean, knowledge about trachoma and the role of personal hygiene, practices such as reducing overcrowding and improving water and sanitation facilities. Targeting of expectant mothers aligns with the habituation strategy of using a life changing event to introduce a novel behaviour. Creating a new community norm for clean faces relates to changing of injunctive norms (i.e., behaviours which are perceived of as being approved/disapproved of by others; these norms assist an individual in determining what is acceptable/unacceptable social behaviour).
- **Monitoring & evaluation methods:** Resource encourages community-wide monitoring of trachoma in a trachoma endemic community until such time as trachoma in no longer a public health problem in the region
    - **Metrics:** Resource suggests monitoring should include both process indicators and outcome measures for hygiene promotion, in particular, regular facewashing. These include training of healthcare workers and clean faces
        - **Types of proposed metrics:** Efficacy of facial cleanliness health promotion materials/activities
        - **Proposed F&E Indicators:** Percentages of children with clean faces, with a target of 80 percent of the children in a community with clean faces, at any given time
- **Tools included:** None

- **Notes:** crowding, poor access to water, failure to use water appropriately, garbage disposal and close proximity of animals as mediators of fly densities

<u>**Women and trachoma: Achieving gender equity in the implementation of SAFE**</u>

Type of document: Technical resource for gender-sensitive implementation of the SAFE strategy, with specific chapters on behaviour change and achieving equity in F&E implementation

- **Purpose of document:** To enable managers and planners of trachoma control programmes to provide a gender-equitable SAFE strategy and ensure that the needs of women are not overlooked
- **Intervention components/activities:** Formative work (e.g., interviews and surveys within participant communities) to elicit information about cultural practices (e.g., taboos, rituals, division of labour) regarding and attitudes toward certain types of F and E-related behaviours, motivation for changing behaviours, and sources of credible information for use in intervention design and implementation; design of contextually-appropriate health education and behaviour change materials with a gender perspective, including pre-testing of draft materials and pilot-testing of the final materials with the target audience; engagement of men to promote a gender-equitable trachoma control programme approach.
    - **Design:** Mixed methods design – qualitative data collection to identify gender-specific attitudes toward and motivators of uptake of improved behaviours; quantitative data collection to determine appropriate communications channels, by sub-group (e.g., men, women)
    - **Do these activities address known barriers:** Resource encourages the employment of interviews and surveys to elicit context-specific information regarding known barriers related to cultural practices and attitudes toward certain types of F and E-related behaviours
- **Implementation approach/strategies:** Resource describes various strategies for gender-sensitive programme approach, such as: 1) identification of programme behaviour change objectives, 2) recognition of gender-specific attitudes toward and motivators of improved behaviours, 3) determination of existing positive behaviours and effective channels of communication within the community – noting similarities and differences between men and women, 4) segmentation of audience to facilitate concurrent targeting of different groups
    - **Type and level of implementation:** Design, implementation **–** community-based design and implementation
    - **Proposed behaviour change frameworks and/or techniques:** Resource provides a general description of the steps to behavioural change, that while not explicitly stated as such, most closely aligns with the Transtheoretical (i.e., Stages of Change) Model. The resource encourages the use of a Positive Deviance Model for health promotion.
- **Monitoring & evaluation methods:**
    - **Metrics:** Resource encourages collection of data regarding attitudes toward certain types of behaviours, motivation for changing behaviours, and perceptions regarding credible sources of information
        - **Types of proposed metrics:** Respondent-reported and/or directly observed behavioural antecedent metrics – individual and community-level
        - **Proposed F&E Indicators:** No specific F&E indicators proposed

- **Tools included:** Appendix C of the document includes tools for conducting background research for health education (i.e., a sample guide for key informant interviews and an example questionnaire to identify information sources and media habits)
- **Notes:** Resource explains how gender defines appropriate social roles, behaviours, and expectations, and thus translates to diverging risks of exposure to trachoma between males and females along their life courses. Important guidance tool with recommendations regarding the identifications of gender norms and using that information to inform gender-sensitive trachoma control programming. Resource includes chapters on behaviour change and achieving gender equity in F and E programming.

<u>The 'ngisipet' and trachoma prevention: solving the latrine problem in nomadic tribes</u>
Type of document: Exchange

- **Purpose of document:** None stated, but inferred purpose is to draw attention to alternative strategies for improving environmental conditions amongst nomadic populations
- **Intervention components/activities:** Producing, promoting, and using the ngisipet, a traditionally styled spade used to bury faeces
    - **Design:** Community-based intervention aimed at improving environmental conditions
    - **Do these activities address known barriers:** Yes, promotion and utilisation of the ngisipet tool, a traditionally styled spade used to bury faeces, acknowledges local taboos and cultural interests, and addresses E-related barriers nomadic populations face, specifically: 1) lack of portability and acceptance of improved latrines, 2) costs associated with the number of latrines that would be required to accommodate a nomadic lifestyle, and 3) latrine construction barriers related to the semi-arid climate and limited access to the tools and resources required to dig and build latrines
- **Implementation approach/strategies:** Community engagement for the development of locally acceptable and culturally appropriate alternatives for improving environmental conditions
    - **Type and level of implementation:** Design and implementation, community level
    - **Proposed behaviour change frameworks and/or techniques:** None specified
- **Monitoring & evaluation methods:** Resource does not discuss monitoring/evaluation methods
    - **Metrics:** None mentioned
        - **Types of proposed metrics:** None mentioned
        - **Proposed F&E Indicators:** None mentioned
- **Tools included:** None

<u>CDNA national guidelines for the public health management of trachoma</u>
Type of document: Technical guidelines for the implementation and monitoring of the national trachoma control programme in Australia

- **Purpose of document:** To provide nationally-consistent guidance to public health units and set a minimum best practice approach for the public health management of trachoma in Australia; provide an evidence base and policy framework for coordinated, community-based activities towards eliminating blinding trachoma from within Aboriginal and Torres Strait Islander communities by 2020 in line with Australia's commitment to the WHO GET2020 initiative

- **Intervention components/activities:** Promotion of facial hygiene to reduce spread of the bacteria (e.g., washing faces as often as is necessary to maintain cleanliness; develop household and personal hygiene skills and behaviours; include facewashing as part of a holistic personal hygiene programme, which may also include handwashing, general hygiene, and tooth-brushing; promote clean faces in children as a norm; empower children, families, and communities to make the physical and behavioural changes necessary to achieve this norm); improvement of environmental conditions (e.g., improve safe access to clean and functioning water supplies; adequate sanitation, including clean linen and aired mattresses; improved housing; reduce overcrowding; minimise fly densities; checking household and community washing facilities to ensure they are functional and safe for children; repairing leaky or broken plumbing; ensuring proper maintenance of bathrooms and laundries; installation or mirrors)
    - **Design:** Community-based engagement in planning, implementation, and evaluation; collaboration from national to local levels for coordination and monitoring
    - **Do these activities address known barriers:** Yes – resource suggests that environmental improvements should focus on reducing the barriers (none specifically mentioned) to children washing their hands and faces and achieving facial cleanliness
- **Implementation approach/strategies:** Ensure engagement with local communities when planning, implementing, and evaluating trachoma programmes[1]; collaboration between public health units, primary health care, and other services working towards trachoma elimination; collection of high quality data to monitor and evaluate progress towards trachoma elimination by improving the coverage, completeness, and timeliness of surveillance data in accordance with the minimum national trachoma dataset
    - **Type and level of implementation:** Planning, implementation, monitoring – local and community levels; reporting to and coordination with relevant national level stakeholders
    - **Proposed behaviour change frameworks and/or techniques:** No specific behaviour change frameworks mentioned, but resource suggests changing norms around clean faces amongst children, changes in physical environment, and development of skills and behaviours
- **Monitoring & evaluation methods:** Resource indicates that ongoing, organised monitoring should be undertaken to assess 'facial cleanliness and environmental health', determine progress, highlight achievements, and provide stakeholders with information about issues that need to be resolved. Resource also provides suggests an 'environmental evaluation' be completed for 'assessing the effectiveness of F&E components' of the national programme.
    - **Metrics:** Resource proposes the measurement of F&E outcome indicators
        - **Types of proposed metrics:** Directly observed, objective measures
        - **Proposed F&E Indicators:** Facial cleanliness, as defined by the absence of nasal and ocular discharge on the face; guidelines suggest a broader environmental assessment of housing, sanitation, waste disposal, and dust control be undertaken to determine whether the infrastructure required to enable facial and hand hygiene is present in home, but the resource does not suggest any specific metrics for doing so.
- **Tools included:** None

[1] The resource notes: "Wherever possible, local knowledge and community priorities should always be considered when implementing the guidelines."

<u>Implementing the SAFE strategy for trachoma control: A toolbox of interventions for promoting facial cleanliness and environmental improvement</u>

Type of document: Technical guidelines for the implementation of interventions promoting F and E

- **Purpose of document:** To provide programme managers and implementers with guidance for designing and implementing interventions for F and E in trachoma control programmes, and to fill a gap in the documentation of the SAFE strategy by giving practical guidelines and highlighting where interventions may or may not work
- **Intervention components/activities:** Resource presents an array of interventions (i.e., 'tools') included in the F&E toolbox along with details related to the basic principles and background regarding the interventions, their respective advantages/disadvantages, situations and locations in which the interventions do/do not work, and case examples (for selected 'tools').
    - **Toolbox interventions:** The following are included in the intervention toolbox: general hygiene promotion, promotion of individual towels and washcloths, school-based trachoma programmes, promotion of water availability, rainwater harvesting, pit latrines, provision of village cleaning equipment
    - **Other interventions that have been tried, and are mentioned in resource:** Other activities that have been tried include: construction of public baths, fly control (e.g., spraying, scatter bait, bottle traps), radio listening clubs, leaky tins, soap-making clubs, and window/door screens
    - **Design:** Community-based interventions that 'generate and maintain momentum' for the design and implementation of F and E interventions
    - **Do these activities address known barriers:** Resource provides guidance on how programme managers should consider and address inhibitors of behaviour change at individual, community, intersectoral, and policy levels
- **Implementation approach/strategies:** Resource highlights the Hygiene Improvement Framework (developed by the Environmental Health Project) as a useful model for planning and implementing F and E interventions for reducing trachoma transmission. In general terms, this framework proposes to work on the following three overarching areas to reduce trachoma transmission: 1) hygiene promotion, including encouraging of existing practices such as face and handwashing, promoting new practices such as individual towel use, and changing key behaviours such as child faeces disposal; 2) access to and utilisation of household and community hardware, including household latrines, community water and waste management systems; and 3) institutional and policy-related enabling environments.
    - **Type and level of implementation:** Design, implementation, monitoring and evaluation – community level
    - **Proposed behaviour change frameworks and/or techniques:** Although resource does not promote any specific behaviour change framework, it does provide a detailed explanation of the general process of behavioural change, motivators and inhibitors of change at various levels (e.g., individual, community, intersectoral, and policy levels). Resource also provides guidance for targeting interventions
- **Monitoring & evaluation methods:** Resource encourages four steps for monitoring and evaluating F and E interventions for trachoma control: 1) developing a purpose and plan for the evaluation, 2) gathering data, 3) analysing data, interpreting findings, and making conclusions and recommendations, and 4) presenting findings and acting on recommendations
    - **Metrics:** While resource does not propose any specific F&E-related process or outcome indicators, it does provide examples of each. Resource also highlights information that should be included in baseline assessments (e.g., indicators related to community's knowledge about hygiene, indicators of behavioural risk factors for trachoma

transmission, indicators related to environmental factors that increase risk of trachoma transmission, motivators of behaviour change, capacity of local health and hygiene professionals)

        - **Types of proposed metrics:** Process, outcome indicators
        - **Proposed F&E Indicators:** Resource does not propose the use of any specific F&E-related indicators, as it suggests the selection of indicators is dependent on the situation. Resource does provide guidance regarding specific considerations that should be taken into account when selecting process and outcome indicators
- **Tools included:** None (not including the toolbox itself)

<u>Pit latrines for all households: The experience of Hulet Eju Enessie Woreda, Amhara National Regional State, Northwest Ethiopia</u>

Type of document: Implementer report

- **Purpose of document:** Inferred purpose was to provide report on the experience of improving latrine coverage in Hulet Eju Enessie woreda, share lessons learnt and best practices
- **Intervention components/activities:**
    - **Design:** Community-based, with government-supported enabling environments
    - **Do these activities address known barriers:** Resource does not highlight any contextually-specific barriers, per se, but the approach to community mobilisation and local political commitment address psycho-social, contextual, and technological barriers. Acknowledging that a lack of latrine coverage was a problem, making it a community and political priority, empowering the community to make a change, and putting the solution in their hands addressed many barriers to the uptake of latrine installation.
- **Implementation approach/strategies:** Community mobilisation (e.g., gaining acceptance and support of the community – acknowledgement that there was poor access to latrines and that this was a problem and a priority for the community to address [i.e., triggering latent demand for sanitation and hygiene facilities]; political leaders working through existing community structures to mobilise people with reference to community practices and cultural norms; conducting a self-sustaining training-of-the-trainer to empower community leaders/early adopters and later community at large to build latrines for themselves, their families, and for demonstration purposes to encourage project participation – political and village leaders leading through example via construction of demonstration latrines; women leveraged [as victims of harmful traditional practices] to petition and motivate their husbands to join the movement; conducting educational sessions on the benefits of latrine ownership and utilisation), strong political commitment amongst local leaders (e.g., linking performance evaluations of political leaders to the success of the latrine construction programme; as a local government objective, leaders were empowered to put sanctions on late-adopters of latrines into place), and integration into pre-existing community structures and practices
    - **Type and level of implementation:** Design, planning, implementation – community-level, with support from local, regional, and federal authorities
    - **Proposed behaviour change frameworks and/or techniques:** No specific behaviour change framework indicated, though working to facilitate change by building skills and capacities
- **Monitoring & evaluation methods:** Resource does not discuss monitoring/evaluation methods
    - **Metrics:** Latrine coverage was used to measure progress amongst local, regional, and federal authorities

        - **Types of proposed metrics:** Unclear whether latrine coverage was reported, confirmed through direct observations, or both
        - **Proposed F&E Indicators:** Latrine coverage, though resource also alludes to latrine use
- **Tools included:** None

<u>The SAFE strategy: Preventing trachoma – A guide for environmental sanitation and improved hygiene</u>

Type of document: Technical guidelines on environmental sanitation and improved hygiene for trachoma prevention

- **Purpose of document:** To address the F and E components of preventive action for trachoma control, for use by community health workers, community leaders, NGOs, and other local stakeholders interested in improving environmental sanitation and perhaps decision-makers in support of hygiene education or the improvement of water supply and sanitation
- **Intervention components/activities:** Manual describes simple methodologies for community participation in control activities, excreta disposal options and their application, methods and materials for hygiene education, methods for vector control, and other measures that can be taken to create a health-promoting environment
    - **Environmental interventions:** Excreta disposal (e.g., installing "proper" latrines that are suitable for fly control [e.g., fly screen, water seal] and encouraging their use, cleanliness, and employing a tightly fitting lid), waste disposal (e.g., isolating solid waste in order to reduce breeding opportunities via designated pit or landfill [consisting of an excavation pit] in which waste is covered with a layer of soil at least once per week), storage of food in fly-proof containers, animal excreta disposal (e.g., stacking dung to reduce surface area and zone in which temperature is suitable for fly breeding, using a light cover [e.g., plastic sheet] on dung heaps or spreading dung on the ground to be dried before flies have time to develop), animal husbandry practices (e.g., keeping animals outside and away from the household living space), use of screens and nets for doors and windows or anti-fly curtains for doorways (e.g., made from strings of beads or strips of plastic) and the use of insecticide on the screens/nets/curtains, use of fly traps (e.g., sticky tapes, lighting traps with electrocutors), use of chemical control methods (e.g., dichlorvos vaporisers, insecticide-impregnated materials or residual insecticides [e.g., viscous paint-on baits] at resting sites), outdoor space spraying (e.g., treatment of breeding sites with larvicides)
    - **Hygiene interventions:** Personal hygiene (e.g., facial cleanliness, handwashing, use of towels or other tissues), hygiene education, general domestic hygiene
    - **Design:** Participatory community-based interventions aimed at environmental improvement and hygiene promotion. Interventions are participatory in that the resource suggests communities should participate in the selection of the most appropriate methods (e.g., for fly control, hygiene education, media channels for communication of messaging)
    - **Do these activities address known barriers:** Resource does not suggest collecting data to tailor F&E-related interventions to context-specific barriers. Instead, the resource encourages the implementation of a prescriptive list of interventions. However, the resource does suggest engaging the community in the selection of interventions such that selected methods are effective, affordable, applicable with locally available materials, simple to implement, adapted to local conditions, and respectful of local beliefs.

- **Implementation approach/strategies:** Participatory selection of interventions intended to address environmental sanitation and improved hygiene for trachoma prevention
  - **Type and level of implementation:** Implementation – community-level
  - **Proposed behaviour change frameworks and/or techniques:** Resource does mention Participatory Hygiene and Sanitation Transformation (PHAST) as a potential method participatory approach to community-based intervention implementation
- **Monitoring & evaluation methods:** Resource does not discuss monitoring/evaluation methods
  - **Metrics:** None mentioned
    - **Types of proposed metrics:** None mentioned
    - **Proposed F&E Indicators:** None mentioned
- **Tools included:** Annex 1 – Chemical methods for fly control

<u>**Teaching series No. 07 - Trachoma**</u>

Type of document: Resource material - teaching slides on trachoma and the SAFE strategy

- **Purpose of document:** Inferred purpose was to increase awareness regarding risk factors of *C. trachomatis* transmission, clinical features of the eye disease through the use of the simplified WHO classification, medical and surgical treatments and preventive measures, including recommendations for personal and community hygiene and sanitation
- **Intervention components/activities:** Establish a community 'contract' to work on trachoma (allows volunteers to be elected and subsequently trained in case recognition and community mobilisation for prevention and control); support a district-level coordinator who coordinates AFE components, trains community health workers, interfaces with village leaders, and collects and disseminated data for programme monitoring; facilitate improvements in community hygiene and sanitation (e.g., regular daily face- and handwashing [encouraging children to remove discharge from their eyes and noses, using a 'leaky tin' to conserve water], ensuring a suitable water supply near the community, keeping flies at a minimum [via designing, installing, and using improved pit latrines; burying or burning rubbish or collective it at a site away from the homestead, housing animals some distance from the homestead], arranging health education within the community with community involvement in the planning and development of such a programme, teaching should be provided for health workers, mothers, children and the community (instruction can be given about trachoma at mother and child health clinics)
  - **Design:** Community-based prevention and treatment implementation and monitoring approach modelled on the SAFE strategy
  - **Do these activities address known barriers:** Resource suggests communities are engaged to identify factors which encourage the spread of transmission – once these are identified, interventions can be designed to bring about environmental and behavioural changes in ways that are acceptable to the respective communities
- **Implementation approach/strategies:** Implementing trachoma control programmes through the district health care system; agreement with the public health framework. Resource suggests that in order to be effective and sustainable, communities need to take part in trachoma control activities, with assistance of members to identify factors which encourage transmission
  - **Type and level of implementation:** Design, implementation, monitoring – local (district) and community levels
  - **Proposed behaviour change frameworks and/or techniques:** No specific frameworks or techniques suggested, though the resource does indicate that health workers and

teachers should be engaged to suggest changes in social and cultural habits and community standards of health care. Resource also indicates the promotion of eye health by education is required to achieve behavioural and environmental changes

- **Monitoring & evaluation methods:** Although the resource highlights a community-based strategy for implementation and monitoring, no specific M&E recommendations were provided
    - **Metrics:** The resource touched on the following indicators, though it does not suggest using them for M&E: time taken to collect water from the primary water source, lack of effective sanitation, inadequate fresh water supplies, presence of animals kept near dwellings and piles of animal dung, overcrowding in homes, presence of nasal and ocular discharge, presence of exposed faeces, rubbish lying in open places
        - **Types of proposed metrics:** No information was provided for how the above-mentioned can/should be measured
        - **Proposed F&E Indicators:** Although the resource discussed the above-mentioned indicators, it did not provide specific guidance on F&E-related indicators
- **Tools included:** Summary of a strategy for implementing a trachoma control programme through the district health care system
- **Notes:** Very limited background is provided for this document. It is not clear who the intended audience consists of, and the purpose of the document was only inferred through a brief and vague introduction. Some of the information provided herein may be outdated, and/or may not reflect current recommendations and/or best practices

<u>Trachoma – a women's health issue</u>

Type of document: Policy/advocacy piece highlighting trachoma as a women's health issue

- **Purpose of document:** Inferred purpose was to increase awareness regarding trachoma as a women's health issue, as the resource indicates all parameters of the disease (its etiology, course, and its medical/surgical, pharmacological, hygiene, and environmental interventions) are either driven or constrained by issues of gender; to advocate for health promotion and education, political will, and effective resource allocation to implement effective interventions
- **Intervention components/activities:** Keeping children's faces clean through facewashing by: 1) addressing the perception that a large quantity of water is needed to wash faces/hands by teaching the importance of facewashing and techniques to minimise the amount of wasted water, 2) working at a community level to ensure all mothers facilitate child facewashing/handwashing practices to address the perception that it is meaningless for some mothers to wash their children's faces if other mothers do not do the same, 3) engaging with men and community leaders to gain legitimacy and authority for changes in water use, 4) facilitating community-level meetings to reinforce strategies that link facewashing to villagers' everyday lives; facilitating community-based health promotion and education regarding the uses of water, sanitation, and waste disposal infrastructure as disease control measures; creating institutional linkages to advocate for and develop policies around investment in basic informational health promotion as a measure for promoting women's health, and in turn, preventing, controlling, and eliminating blinding trachoma
    - **Design:** Community engagement to promote facewashing and education regarding the uses of water and sanitation as disease control measures; cross-sectoral collaboration
    - **Do these activities address known barriers:** Yes – the proposed interventions address known barriers surrounding behavioural antecedants regarding knowledge, attitudes,

        perceptions, and norms regarding F&E-related practices. Resource does not suggest an assessment of context-specific barriers, per se

- **Implementation approach/strategies:** Cross-sectoral collaboration to enhance investment in community-based interventions for trachoma prevention and control via a women's health lens
    - **Type and level of implementation:** Cross-sectoral collaboration for advocacy/policy implementation (global/national levels); implementation of preventive interventions (community level)
    - **Proposed behaviour change frameworks and/or techniques:** No specific behaviour change framework or techniques highlights, though the author does indicate the importance of addressing **social norms** regarding legitimacy and authority for making changes to household and community level water use and wide-spread washing of children's faces (women studied indicated it was meaningless to wash their children's faces if other mothers did not do the same), **perceptions** about the amount of water available and household priorities for water use
- **Monitoring & evaluation methods:** Resource does not discuss monitoring/evaluation methods
    - **Metrics:** Although no specific metrics were suggested for monitoring, the authors did mention the need to address barriers related to certain knowledge, attitudes, perceptions, and norms related to F&E practices and the causes of ill health, more generally
        - **Types of proposed metrics:** None mentioned
        - **Proposed F&E Indicators:** None mentioned
- **Tools included:** None
- **Barriers highlighted:** Lack of awareness that trachoma is preventable and that treatment can halt progression toward irreversible damage (social norms in many patriarchal societies require women to be stoic and uncomplaining about ill health), social norms regarding allocation of water resources (men to legitimise water use), varying beliefs about the causes of ill health (e.g., old age, evil eye, discussion of the disease is equal to questioning the will of God), perceptions regarding the amount of water available and households priorities for use of water

Achieving community support for trachoma control: A guide for district health work

Type of document: Technical guidelines for district health workers to cultivate community support for and encourage community action toward trachoma control activities – full SAFE strategy covered

- **Purpose of document:** To provide district health workers with guidelines for: 1) achieving community support for trachoma control activities; and 2) detecting, treating, and preventing the disease. Authors indicate the manual can be used in a number of ways to stimulate community interest in preventing blindness from trachoma, serving as a tool to guide: 1) learning about trachoma and training of others, 2) investigation of trachoma in the community, and 3) generation of practical ideas for how to achieve community support for trachoma control
- **Intervention components/activities:** ***F-related activities highlighted in the resource include***: increasing the number of times a child washes his/her face (i.e., removing discharge from the face so flies are not attracted to the face, teaching older siblings to increase facewashing of younger brothers and sisters, encouraging primary and pre-primary schools to promote facewashing, involving men in all efforts to keep children's faces clean). Resource indicates required inputs related to personnel (a. those to engaging in keeping children's faces clean: e.g., women, mothers, grandmothers, fathers, brothers and sisters; b. those to engage in promoting facewashing: e.g.,

everyone, community health workers, school teachers and children, traditional healers, traditional birth attendants), administration, equipment (e.g., leaky tins, gourds, or pitchers), supplies (e.g., materials for use by school teachers), and transport. ***E-related activities highlighted in the resource include:*** 1) changes at the community level (e.g., local health workers and communities identifying village development priorities and work for change; improving water supply, sanitation, and housing by establishing links with people and organisations who can help plan water and sanitation projects; local health workers and communities identifying what, if anything, can diminish flies breeding in cow dung); and 2) changes at the household level (e.g., households constructing ventilated, improved latrines and using them to reduce the number of flies; disposing of rubbish to minimise fly breeding sites; separating and ventilating sleeping areas). Resource indicates required inputs related to personnel (a. those to engage in advocating for improved environment: e.g., functional and active committees or groups working in the area such as health committees, community leaders, public works, politicians, NGOs; b. those to engage in supporting local community participation: development workers, women's groups, village health workers), training (e.g., establishing and maintaining effective communities committees, making toilets, constructing water jars), supplies (e.g., materials for construction), transport (e.g., vehicles to transport materials and to go to meetings)

  - **Design:** Participatory, community-based implementation
  - **Do these activities address known barriers:** Yes, the participatory, community-based approach proposed herein encourages local health workers to work with communities to identify their specific constraints to F&E while also providing general guidance that addresses barriers to perceptions regarding the amount of water necessary for facewashing and finding the time to wash children's faces often, knowledge regarding the benefits of keeping children's faces clean, attitudes regarding construction and utilisation of latrines, decision-making authority at village and household levels, communication between communities and development workers, and a shortage of skills in the village. Proposed solutions for F-related barriers include: changing perceptions regarding the amount of water and time necessary for facewashing, promoting of habituation of regular facewashing through as many channels as possible (e.g., mothers, women's groups, churches, primary schools, adult literacy classes, village health workers); and encouraging mother's discussion groups regarding strategies for increasing facwashing

- **Implementation approach/strategies:** Resource suggests the employment of a participatory community-based approach to learn from the community what they are willing and interested in doing to prevent trachoma and how these interventions can be integrated into existing health, education, religious, and social activities in the village
  - **Type and level of implementation:** Design, planning, implementation – community level
  - **Proposed behaviour change frameworks and/or techniques:** Perceptions regarding priorities (women) and the amount of water necessary for facewashing mentioned; attitudes regarding constructing and using latrines; knowledge regarding the benefits of facial cleanliness and how flies transmit trachoma mentioned; decision-making authority regarding changes in the home, changes in the community; history of collective action in the community; hardware and utilisation skills at household level: how to keep faces clean with little water, what can be done to reduce flies, constructing water storage tanks and toilets; behavioural changes at household and village levels related to keeping household and village environments clean
- **Monitoring & evaluation methods:** Resource does not discuss monitoring/evaluation methods
  - **Metrics:** Although no specific metrics were suggested for monitoring, the authors did mention it was important to engage the community in assessing the following indicators:

lack of water (e.g., how far do people go to fetch water, who decides how the water will be used, how much water do people think is needed to keep children's faces clean), a lot of flies, overcrowding where children sleep, smoke from a cooking fire, dust,
        - **Types of proposed metrics:** None mentioned
        - **Proposed F&E Indicators:** None mentioned
- **Tools included:** Discussion guide for facilitating conversations with the community about the various components of the SAFE strategy – F&E-related questions focus around whether people will accept facewashing to keep childiren's faces clean and whether people will make environmental improvements in their households and villages

General WASH-NTD grey literature

Water, sanitation, and hygiene for accelerating and sustaining progress on NTDs: A global strategy 2015-2020

- **Type of document:** Technical guidance for joint cross-sectoral/programme planning, implementation, and evaluation of activities to achieve common goals of WASH and NTD initiatives
- **Purpose of document:** Strategic plan to mobilise WASH and NTD actors to work together toward NTD roadmap targets. The strategy calls on WASH funders and implementers to target NTD endemic areas and deliver programmes that maximise the effectiveness of WASH interventions for NTD control and elimination
- **Intervention components/activities:** Linking WASH and NTDs to impact multiple NTDs through a single area of intervention. Plan includes 3 specific objectives: 1. Increase awareness about the co-benefits of joint WASH and NTD action by sharing experiences and evidence from improved delivery; 2. Use WASH and NTD monitoring to highlight inequalities, target investment, and track progress; 3. Strengthen evidence on how to deliver effective WASH interventions for NTDs and embed the findings in guidance and practice; and 4. Plan, deliver and evaluate programmes with mutual inputs from WASH, health and NTD stakeholders at all levels.
    - **Design:** No specific guidance was provided on the design of intervention components, though the following activities were suggested, and illustrative examples provided:
        - **Objective 1:** Identify synergies across NTDs, and between NTDs and WASH; Strengthen platforms for sharing knowledge and increasing collaboration; Improve awareness about NTDs and opportunities provided by joint interventions among professional communities within and beyond WASH and NTDs
        - **Objective 2:** Formulate cross-cutting programme monitoring and evaluation systems, including standardized and comparable success indicators at global, national and subnational levels; Collect higher quality and more disaggregated data, and developing monitoring capacity
        - **Objective 3:** Define an agenda for applied, practical operational research on effective implementation in practice for research institutions and implementers; Embed guidance on joint implementation for NTD control in WASH standards and guidelines, and dissemination and uptake of standards and guidelines into policy and practice; Embed guidance on collaboration with WASH programmes in NTD standards and guidelines

        - **Objective 4:** Support the development and strengthening of governance and institutional arrangements; Joint use of existing datasets and reports between stakeholders and across sectors to track progress and inform decision-making; Develop and use of new and existing integrated planning tools; Create and support financial arrangements that enable collaboration across the NTDs and with WASH; Establish a framework for planning personal hygiene behaviour change aspects for both control and disability aspects of NTD programmes
    - **Do these activities address known barriers:** Yes. Addressing barriers to and facilitators of behaviour change are often overlooked during programme design, planning, and implementation processes. This plan suggests that strategies to address these barriers should be informed by formative research and consultation, and incorporated into planning
- **Implementation approach/strategies:** Creating and fostering cross-sectoral partnerships and collaboration
    - **Type and level of implementation:** Planning – Global (WHO) and national levels
    - **Proposed behaviour change frameworks and/or techniques:** Creation (and execution) of a framework for personal hygiene behaviour change
- **Monitoring & evaluation methods:** See objective 2 above
    - **Metrics:** No specific metrics outlined
        - **Types of proposed metrics:** Haromonised M&E frameworks, inclusion of process indicators to monitor the development and implementation of policy and governance frameworks to enable coordination
        - **Proposed F&E Indicators:** N/A – none provided, but the document is not trachoma-specific
- **Tools included:**
    - Action plan (for WHO and endemic countries/their partners)
    - The role of WASH in NTD prevention and care
- **Notes:** This document is linked to the following peer-reviewed publication: Boisson S, Engels D, Gordon BA, Medlicott KO, Neira MP, Montresor A, Solomon AW, Velleman Y. Water, sanitation and hygiene for accelerating and sustaining progress on neglected tropical diseases: a new Global Strategy 2015-20. *Int. Health*. 2016; 8(suppl 1): i19-121

<u>**WASH and the neglected tropical diseases: A global manual for WASH implementers**</u>

- **Type of document:** Technical guidance for WASH implementers to address 5 key NTDs through collaborative monitoring of NTD-specific health outcomes and impacts to inform programme and policy change
- **Purpose of document:** To enable WASH practitioners who work at the country level to contribute to the reduction of WASH-preventable NTDs
- **Intervention components/activities (trachoma-specific):** promote regular facewashing with soap to remove eye and nasal discharge potentially contaminated with bacteria; encourage regular washing of clothing and bedding with soap to prevent further spread of bacteria; reduce open defecation to remove breeding sites for eye-seeking flies; increase access to and use of household latrines; promote latrine maintenance and cleaning, which facilitates latrine use by all members of the family and prevents accumulation of faeces in the open, which can attract flies; improve access to sufficient amounts of water, which can lead to increased water use for household hygiene practices (e.g., washing hands, face, body; bathing; doing laundry) and environmental

sanitation (e.g., cleaning latrines); dispose of faeces properly, including but not limited to infant/child faeces disposal

    - **Design:** Resource encourages integration of trachoma-specific messages into existing hygiene education (e.g., washing of faces, soiled clothing, and bedding) and collaborative monitoring of disease outcomes
    - **Do these activities address known barriers:** Resource does not provide recommendations for addressing barriers to improve WASH behaviours
- **Implementation approach/strategies:** Integration of WASH-NTD messages, collaboration via joint monitoring of trachoma-specific health outcomes and impacts to inform programmatic and policy change
    - **Type and level of implementation:** Integration and collaboration at national to community levels
    - **Proposed behaviour change frameworks and/or techniques:** Resource does not mention any specific frameworks or techniques
- **Monitoring & evaluation methods:** Resource encourages post-implementation monitoring of water services, sanitation systems, and hygiene behaviour to assess sustainability of outcomes. Resource highlights the possibility that WASH-preventable NTDs that are monitored regularly may serve as sentinel indicators of the functionality of WASH services, and measuring the reduction in NTD prevalence and health gains allows for an assessment of the impacts of WASH services.
    - **Metrics:** Resource mentioned F&E-specific indicators
        - **Types of proposed metrics:** Outcome indicators, no specification regarding respondent-reported vs. directly observed and/or objective indicators
        - **Proposed F&E Indicators:** facewashing, facial cleanliness, access to household latrines, access to water supplies
- **Tools included:**
    - Monitoring and evaluation summary to help WASH practitioners collaborate with other stakeholders to jointly measure programme impact on NTDs
    - Advocacy messaging tool
    - Policy landscape for NTD control resources

WASH: The silent weapon against NTDs

Type of document: Policy/advocacy piece for better WASH-NTD integration at global to community levels

- **Purpose of document:** None stated, but inferred purpose is to articulate why water, sanitation, and hygiene matter for sustainable elimination of NTDs; to place a call to action for better WASH-NTD integration at global to community levels and improved sharing of knowledge regarding current WASH best practices that facilitate sustained adoption of improved NTD-preventive WASH behaviours
- **Intervention components/activities:** Identifying practical opportunities for integration; cross-sectoral collaboration, including developing local and global partnerships, sharing of information and evidence regarding the disease impacts of WASH-related NTD preventive interventions, jointly advocating for resources and political commitment to action, planning sustainable programmes from the outset that are designed to meet goals for sustainable disease elimination and WASH
    - **Design:** Resource encourages the design and implementation of community-based, context-specific demand-side or combined demand and supply-side interventions that are informed by formative research; collaborative cross-sectoral partnerships for integrative programme at global to local levels

    - **Do these activities address known barriers:** Yes – community-based, demand-driven or combined demand and supply-side interventions address barriers to uptake of improved WASH practices related to affordability, preferences, and demand
- **Implementation approach/strategies:** Resource highlights increasing awareness within the WASH sector of the need to move from thinking about programme implementation to a Service Delivery Approach (SDA) and sustainable behaviour change; creating and fostering cross-sectional partnerships; and design/implementation of demand-side interventions (e.g., community-led total sanitation [CLTS]) that empower communities to take collective action to stop open defecation or combine demand-side and supply-side measures to generate widespread sanitation demand and increase the supply of sanitation products and services at scale (e.g., total sanitation and sanitation marketing [TSSM])
    - **Type and level of implementation:** Design, planning, implementation – Global to community levels
    - **Proposed behaviour change frameworks and/or techniques:** Resource highlights WaterAid's Sanitation Framework which references nine sanitation principles that should underpin any sanitation approach. This framework explains that supply-driven, subsidised delivery of sanitation (and hygiene) infrastructure is often unwanted, expensive, and does not meet user preferences, thus resulting in a lack of sustainable adoption of improved behaviours and practices. Resource also advocates for the shift from hygiene education to hygiene promotion based on formative research aimed at elucidating the motivators of behaviour change and fostering of opportunities and abilities needed to put new behaviours into practice.
- **Monitoring & evaluation methods:**
    - **Metrics:** Resource does not discuss specific F&E-related M&E metrics, though it does advocate for alignment of indicators with sector M&E frameworks, country-level information systems, or the NTD Global Milestone Scorecards
        - **Types of proposed metrics:** No specific metrics proposed
        - **Proposed F&E Indicators:** No specific F&E indicators proposed
- **Tools included:** Resource does not include additional tools, per se, but does highlight nine sanitation principles and ten practical opportunities for WASH-NTD integration
- **Notes:** Resource highlights that people rarely want sanitation for reasons of health. This principle draws on a considerable evidence base that suggests health considerations are not effective in changing behaviour.

Other grey literature

Handbook on community-led total sanitation (CLTS)

Type of document: Technical guidance for the implementation of CLTS

- **Purpose of document:** To bring together experience, diversified practice and local innovations from different countries and many sources to meet the need and demand for a source that provides guidance on pre-triggering, critical post-triggering activities, and scale up
- **Intervention components/activities:** Community awareness raising and mobilisation via pre-triggering, triggering, and post-triggering activities, including: rapport building, facilitating community appraisal and analysis, conducting transect walks, mapping of defecation areas, calculating shit and medical expenses, facilitating the community's plan of action, using

community mapping for monitoring, following-up and encouragement of community mobilisaiton, engaging existing community mechanisms, verifying and certifying ODF status, sustaining and monitoring sustained ODF status

- **Design:** Participatory, community-based interventions aimed at abolishing open defecation. Of note, while CLTS does seek to improve general hygiene, the traditional approach does not emphasise specific F-related interventions or metrics
- **Do these activities address known barriers:** Demand-side sanitation approaches address issues regarding barriers to uptake of sanitation facilities related to affordability and user preferences. That said, this resource does not promote the collection of data on other contextually-specific barriers to inform the design of the demand-side intervention or its implementation, so depending upon the implementation approach, CLTS may not necessarily address the array of context-specific barriers

- **Implementation approach/strategies:** Community-based, participatory approach; stimulating latent demand and/or demand creation; cooperative approach that focuses on the collective benefit of stopping open defecation by concentrating on changing behaviours of the whole community as opposed to individuals
    - **Type and level of implementation:** Implementation and support, monitoring – community-based
    - **Proposed behaviour change frameworks and/or techniques:** Use of psycho-social drivers such as disgust to trigger a change sanitation behaviour
- **Monitoring & evaluation methods:** Resource encourages the use of a community map that displays each household and indicates whether the household has 'gained access to a latrine' and are using the latrine as a means of participatory community monitoring. Resource also suggests engaging community members and natural leaders in the identification of other indicators they wish to use to monitor the community's progress toward open defecation, such as those indicated under 'metrics'
    - **Metrics:** Individual, household, community level process and outcome indicators identified by the community, such as: number and range of natural leaders who emerge, from women, men, youth, and others; households constructing latrines jointly; sharing latrines between neighbours or relatives or traditional or other groupings; formation of new groups; better off people coming forward to help those who are weaker and poorer; revival of traditional communal cooperation groups; volunteers, traditional midwives, and others becoming active; new slogans, songs, and poems; increased sales of sanitary hardware in markets; the emergence of community sanctions against open defecation; communities providing collective help to those with difficulties; sanitary hardware given as gifts (e.g., to newly married couples); reduction in diarrhoeal diseases and in sales of medicines for such diseases; decreasing health expenditure and income of village quacks; fewer flies
        - **Types of proposed metrics:** Respondent-reported, directly observed, objective measures
        - **Proposed F&E Indicators:** Resource does not propose any F-related metrics, but suggested E-related metrics include: indicators of latrine use (e.g., well-worn path to the latrine, "other" latrine attributes/spot check indicators), distinct and visible indicators of hygiene behaviour change (e.g., soap for handwashing, water containers near latrine), continued presence of hanging/floating latrines and/or other open defecation sites
- **Tools included:** Appendix C: Triggering in special conditions, Appendix D: Sample questions for raising disgust, Appendix E Sample Checklist of follow-up activities
