# Supplementary figures and images for: Interventions to maximize facial cleanliness and achieve environmental improvement for trachoma elimination: A review of the grey literature
Source: PLoS Negl Trop Dis. 2018 Jan 25;12(1):e0006178. doi: 10.1371/journal.pntd.0006178 (PMC5800663; doi:10.1371/journal.pntd.0006178)

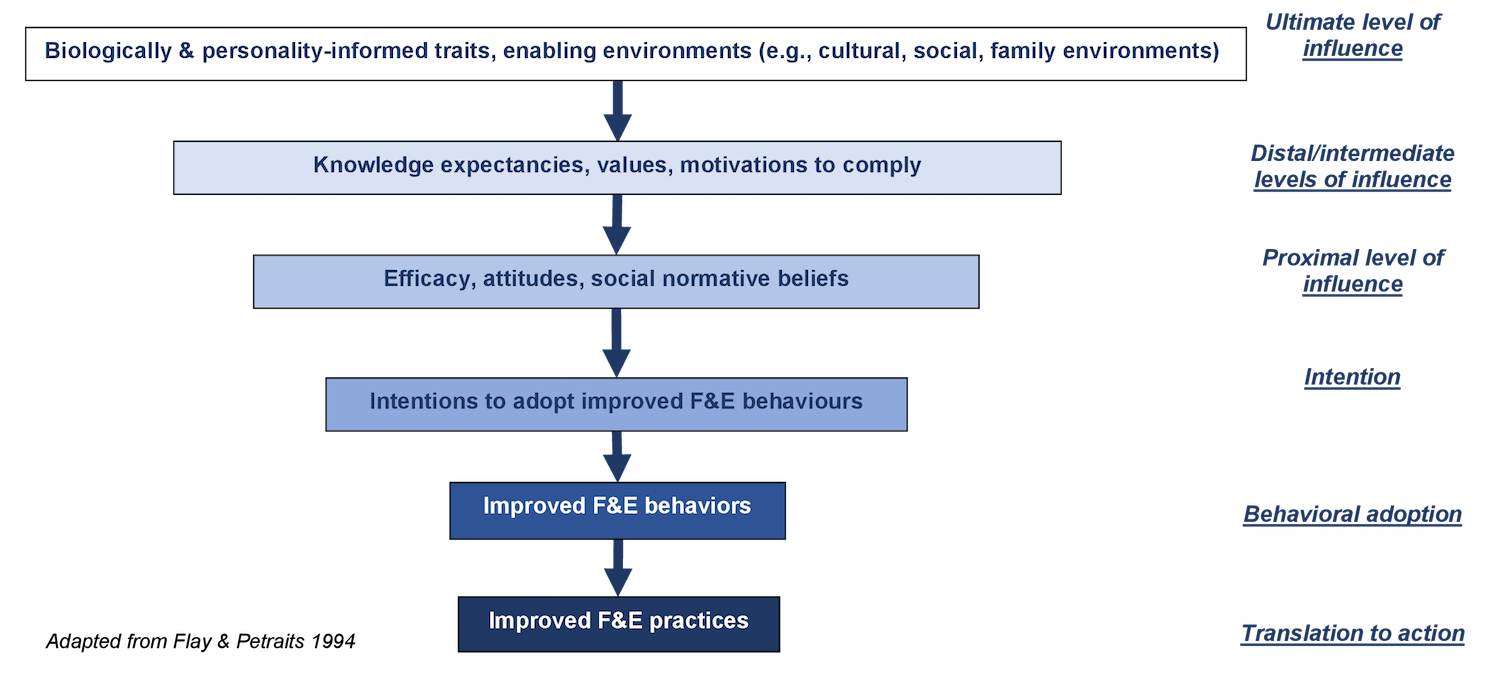

Supplement: S1 Fig — (TIF) [file pntd.0006178.s004.tif]
