# Supplementary material for: Interventions to maximize facial cleanliness and achieve environmental improvement for trachoma elimination: A review of the grey literature
Source: PLoS Negl Trop Dis. 2018 Jan 25;12(1):e0006178. doi: 10.1371/journal.pntd.0006178 (PMC5800663; doi:10.1371/journal.pntd.0006178)
Supplement: S1 Table — (PDF) [file pntd.0006178.s005.pdf]

S1 Table. Example* Boolean search strategy employed during electronic searches of grey literature databases

SEARCH SET	DESCRIPTION	SEARCH STRATEGY+
1.	Facial cleanliness	(clean face or facial clean* or dirty face or nasal discharge or ocular discharge or fly-eye or fly eye or ((face OR facial) AND debris) or face wash* or facewash* or face-wash* or bath* or wipe OR wiping or wash* or hand wash* or handwash* or hygiene or wash cloth* Or washcloth* or towel*) AND trachoma*
2.	Environmental improvement	((environment* improve*) OR sanita* OR faeces OR feces OR faecal OR fecal OR excre* OR waste OR defecat* OR latrine OR toilet OR water OR flies OR sorbens OR Bazaar fly OR Bazaar flies OR ((cattle OR animal husbandry) AND trachom*) OR ((crowd* OR crowds OR crowded OR crowding OR population density) AND trachom*)) AND trachoma*
3.	Facial cleanliness behaviour change	(clean face or facial clean* or dirty face or nasal discharge or ocular discharge or fly-eye or fly eye or ((face OR facial) AND debris) or face wash* or facewash* or face-wash* or bath* or wipe OR wiping or wash* or hand wash* or handwash* or hygiene or wash cloth* Or washcloth* or towel*) AND (“behaviour change” OR “behavior change” OR “behavioural change” OR “behavioral change” OR “changing behaviour” OR “changing behavior” OR “behavioural framework” OR “ behavioral framework” OR “behaviour framework” OR “behavior framework” OR “behavioural theory” OR “ behaviour theory” OR “behavioral theory” OR “behavior theory” OR “theory of change” OR “behaviour change theory” OR “behavior change theory” OR “behavioural change theory” OR “behavioral change theory” OR “hygiene education” OR “health education”) AND trachoma*
4.	Environmental improvement behaviour change	((environment* improve*) OR sanita* OR faeces OR feces OR faecal OR fecal OR excre* OR waste OR defecat* OR latrine OR toilet OR water OR flies OR sorbens OR Bazaar fly OR Bazaar flies OR ((cattle OR animal husbandry) AND trachom*) OR ((crowd* OR crowds OR crowded OR crowding OR population density) AND trachom*)) AND (“behaviour change” OR “behavior change” OR “behavioural change” OR “behavioral change” OR “changing behaviour” OR “changing behavior” OR “behavioural framework” OR “ behavioral framework” OR “behaviour framework” OR “behavior framework” OR “behavioural theory” OR “ behaviour theory” OR “behavioral theory” OR “behavior theory” OR “theory of change” OR “behaviour change theory” OR “behavior change theory” OR “behavioural change theory” OR “behavioral change theory” OR “hygiene education” OR “health education”) AND trachoma*

NOTES:
* This is an illustrative example of what was set out in the review protocol. Different electronic databases accommodate different search parameters. As such, actual Boolean searches may have differed slightly, by database.
+ As indicated in our inclusion criteria, we employed limitations to limit returned publications to those meeting language and year requirements
