# Supplementary material for: Interventions to maximize facial cleanliness and achieve environmental improvement for trachoma elimination: A review of the grey literature
Source: PLoS Negl Trop Dis. 2018 Jan 25;12(1):e0006178. doi: 10.1371/journal.pntd.0006178 (PMC5800663; doi:10.1371/journal.pntd.0006178)
Supplement: S2 Table — (PDF) [file pntd.0006178.s006.pdf]

S2a Table. Key word search terms (outlined in review protocol)

KEY WORD	RELATED SEARCH TERMS
**‘F and E’**	
	Facial cleanliness and environmental improvement
	‘F and E’
	‘F & E’
**Facial cleanliness**	
	Facial cleanliness
	Clean face
	Dirty face (antonym)
	Nasal discharge
	Ocular discharge
	Facial debris, debris on face
	Fly-eye contacts, fly eye contact
	Facewash, face wash, face-wash
**Personal hygiene**	
	Personal hygiene, hygiene, hygienic
	Bathing, bathe, bath
	Washing, wash
	Wiping, wipe, towel, wash cloth
	Handwash, hand wash, hand-wash
**Environmental improvement**	
	Environmental improvement
	Water
	Sanitation
	Defecation, open defecation
	Latrine, toilet
	Faeces, faecal, feces, fecal, excreta
	Musca sorbens, *M. sorbens*, flies, fly
	Bazaar fly, Bazaar flies
	Fomites
	Waste, human waste, animal waste, solid waste, liquid waste
	Cattle, cattle keeping, cattle herding, animal husbandry
	Crowding, population density
**Behavior change**	
	Behaviour change, behavior change, changing behaviour, changing behavior
	Behavioural framework, behavioral framework, behavioural theory, behaviour theory, behavioral theory, behavior theory
	Theory of change
	Hygiene education, health education
	Uptake, adoption, habituation
	Social norms, social network, social influence, social learning

S2b Table. Key word search terms (employed for systematic website document identification)

KEY WORD	RELATED SEARCH TERMS
**Trachoma**	
	'trachom', trachom
	'trachoma', trachoma
**'F and E'**	
	Facial cleanliness and environmental improvement, 'facial cleanliness and environmental improvement'
	'F and E', F and E
	'F & E', F & E, 'F&E', F&E
	'Facial cleanliness', facial cleanliness
	'Environmental improvement', environmental improvement
**Personal hygiene**	
	'hygiene', hygiene
**Behavior change**	
	'Behaviour change', behaviour change
	'Behavior change', behaviour change
