# Supplementary material for: Interventions to maximize facial cleanliness and achieve environmental improvement for trachoma elimination: A review of the grey literature
Source: PLoS Negl Trop Dis. 2018 Jan 25;12(1):e0006178. doi: 10.1371/journal.pntd.0006178 (PMC5800663; doi:10.1371/journal.pntd.0006178)
Supplement: S3 Table — (PDF) [file pntd.0006178.s007.pdf]

S3 Table. Antecedent behavior change factors addressed in the grey literature				
Behavioral factor*	Description*	Stated by authors of reference	Inferred by reviewers	AGGREGATE
**RISK FACTORS - UNDERSTANDING AND AWARENESS OF RELATED HEALTH RISKS**				
**Perceived vulnerability**	Subjective perception of the risk of contracting trachoma	3	0	3
**Perceived severity**	Subjective perception of the seriousness of the consequences of active trachoma/trichiasis	0	0	0
**Health/hygiene knowledge**	Knowledge about a disease's causes and (personal) consequences and its preventive measures	16	5	21
Ill-health/good health beliefs	Perceived reasons for ill/good health, including spiritual, cultural, biological causes	2	1	3
**Total RISK FACTOR citations amongst reviewed literature**				27
**ATTITUDE FACTORS - POSITIVE OR NEGATIVE STANCE TOWARD A PARTICULAR BEHAVIOR OR SET OF BEHAVIORS**				
**Cost beliefs**	Perceived negative aspects of engaging in improved F&E-related practices	3	5	8
**Benefit beliefs**	Perceived positive aspects of engaging in improved F&E-related practices	3	10	13
**Affective beliefs/feelings**	Beliefs concerning feelings associated with performing improved F&E-related practices; one's emotions (e.g., joy, pride, disgust) which arise when thinking of/carrying out improved F&E-related practices or consequences thereof	0	5	5
**Total ATTITUDE FACTOR citations amongst reviewed literature**				26
**NORMATIVE FACTORS - PERCEIVED SOCIAL PRESSURE TOWARDS A BEHAVIOR; OTHER'S ACTIONS AND OPINIONS REGARDING A BEHAVIOR OR SET OF BEHAVIORS**				
**Others' behaviors**	Observation and awareness of other's behaviors and practices; perceptions as to which behaviors and practices are typically carried out by others (i.e., empirical beliefs, descriptive norms)	2	3	5
**Others' (dis)approval**	Perceptions regarding behaviors typically (dis)approved of by relatives, friends, community (i.e., normative beliefs, social norms)	13	1	14
**Personal importance**	Beliefs regarding what one ought or ought not to do	0	0	0
**Total NORMATIVE FACTOR citations amongst reviewed literature**				19
**ABILITY FACTORS - CONFIDENCE IN ONE'S ABILITY TO PRACTICE A BEHAIVOR**				
**Action knowledge**	Knowledge about how to act on improved F&E-related behaviors (execute or put a behavior into practice)†	0	10	10
Action capacity	Skills, access to resources required to put improved F&E-related behaviors into practice	12	8	20
**Self-efficacy/confidence in performance‡**	Belief in one's personal capacities to perform the practices necessary to produce improved F&E-related performance attainments§	3	2	5
**Confidence in continuation**	Perceived ability to continue to practice a behavior, which includes the person's confidence in being able to deal with barriers that may arise	0	0	0
**Confidence in recovering**	Perceived ability to recover from setbacks, to continue the behavior after disruptions	0	0	0
**Total ABILITY FACTOR citations amongst reviewed literature**				35

SELF-REGULATION FACTORS - ATTEMPTS TO PLAN AND SELF-MONITOR A BEHAVIOR AND TO MANAGE CONFLICTING GOALS AND DISTRACTING CUES				
**Action planning**	Specification of when, where, and how to perform improved F&E-related practices	**0**	**5**	**5**
**Action control**	Self-monitoring and effort to continuously evaluate on-going improved F&E-related practices	**1**	**0**	**1**
**Barrier planning**	Extent to which one attempts to plan to overcome barriers which would impede the adoption/execution of improved F&E-related behaviors and/or related practices	**1**	**3**	**4**
**Remembering**	Perceived ease of remembering to practice the improved F&E-related behavior in key situations	**1**	**0**	**1**
**Commitment**	Obligation one feels to practice the improved F&E-related behaviors	**4**	**1**	**5**
Habituation of improved practices	F&E-related practices that are triggered automatically in response to contextual cues that have been associated with their performance[ǁ]	**4**	**4**	**8**
Reinforcement of behaviors & practices	Use of rewards (e.g., recognition, praise) to encourage one to carry out improved F&E-related behaviors and practices	**3**	**8**	**11**
**Total SELF-REGULATION FACTOR citations amongst reviewed literature**				**35**

NOTES: Reviewers utilized the RANAS approach to systematic behavior change [11] as the foundation for the review of F&E-related interventions endorsed in the grey literature. Italicized text indicates factors added by authors, based on their inclusion in other related theoretically-grounded and empirically-supported behavior change frameworks. Totals related to action capacity include 12 documents addressing skills required, while the remaining 8 documents address resources required to perform improved F&E practices.

*Adapted from Mosler 2012 [12]

† See Cohn 2014 [14]

‡ Self-efficacy is an important determinant of personal, and perhaps household-level behavior change. Authors of the review suggest that collective efficacy is an important determinant of community-level behavior change, particularly for community-based interventions

§ Adapted from Bandura, A. 1977 [28]

ǁ Neal, D. 2012 [64]
