# Supplementary material for: Interventions to maximize facial cleanliness and achieve environmental improvement for trachoma elimination: A review of the grey literature
Source: PLoS Negl Trop Dis. 2018 Jan 25;12(1):e0006178. doi: 10.1371/journal.pntd.0006178 (PMC5800663; doi:10.1371/journal.pntd.0006178)
Supplement: S4 Table — (PDF) [file pntd.0006178.s008.pdf]

S4 Table. Conceptual and behavioral frameworks indicated in the grey literature	
**Brief description of conceptual/behavioral framework**	**Reference**
**Integrated Behavioural Model for Water, Sanitation, and Hygiene (IBM-WASH)**<br>A behavior change model that acknowledges 3 dimensions (contextual, psychosocial, technology factors) that operate on 5 levels (structural, community, household, individual, habitual)	Formative research assessment and guidelines: Facial cleanliness and environmental sanitation
**Communication for Water Treatment & Hygiene Framework**<br>A predictive model developed to guide the design & evaluation of interventions that promote improved water, sanitation, & hygiene behaviours by linking outcome evidence to specific health communication strategies	• Formative research assessment and guidelines<br>• Understanding individual and contextual factors for development of a behavior change communication campaign for trachoma prevention in Busoga and Karamoja regions, Uganda
**Risk Perception Attitude Framework (RPA)**<br>A framework adapted from the Extended Parallel Process Model (EPPM) that explains the mediating role of self-efficacy in the relationship between risk and behaviors	Understanding individual and contextual factors for development of a behavior change communication campaign for trachoma prevention in Busoga and Karamoja regions, Uganda
**Evo-Eco approach to behaviour change**<br>A behavior change approach with a foundation in evolutionary biology & ecological psychology that suggests brains evolved to provide adaptive behavioral responses to rapidly changing or complex environmental conditions	Research to inform the development of behavior change interventions for "F" and "E" of the SAFE strategy in Turkana and Marsabit, Kenya
**Positive Deviance (PD) approach**<br>A strengths-based, problem solving approach to behavioral & social change that enables a community to discover existing solutions to complex problems within the community	Women and trachoma: Achieving gender equity in the implementation of SAFE
**Hygiene Improvement Framework** (developed via the Environmental Health Project)<br>Comprehensive approach to hygiene improvement that addresses three elements: hygiene promotion, access to hardware, and support from enabling environments	Implementing the SAFE strategy for trachoma control: A toolbox for promoting facial cleanliness and environmental improvement
**Participatory Hygiene and Sanitation Transformation (PHAST)**<br>A participatory learning approach that aims to help communities improve hygiene behaviors and encourage effective management of water and sanitation services	The SAFE strategy: Preventing trachoma - A guide for sanitation and improved hygiene
**Community-led total sanitation (CLTS)**<br>A demand-side approach that mobilizes communities to completely eliminate open defecation through the "triggering" of latent demand and/or creation of novel demand for access to and utilization of sanitation facilities	Handbook on community-led total sanitation
